# PRMT4 Is a Novel Coactivator of c-Myb-Dependent Transcription in Haematopoietic Cell Lines

**DOI:** 10.1371/journal.pgen.1003343

**Published:** 2013-03-07

**Authors:** Gundula Streubel, Caroline Bouchard, Hannah Berberich, Marc S. Zeller, Sophia Teichmann, Jürgen Adamkiewicz, Rolf Müller, Karl-Heinz Klempnauer, Uta-Maria Bauer

**Affiliations:** 1 Smurfit Institute of Genetics, Trinity College Dublin, Dublin, Ireland; 2 Institute for Molecular Biology and Tumor Research (IMT), University of Marburg, Marburg, Germany; 3 Centre de Regulació Genòmica (CRG) and UPF, Barcelona, Spain; 4 Institute for Biochemistry, Westfälische Wilhelms-University of Münster, Münster, Germany; Stanford University School of Medicine, United States of America

## Abstract

Protein arginine methyltransferase 4 (PRMT4)–dependent methylation of arginine residues in histones and other chromatin-associated proteins plays an important role in the regulation of gene expression. However, the exact mechanism of how PRMT4 activates transcription remains elusive. Here, we identify the chromatin remodeller Mi2α as a novel interaction partner of PRMT4. PRMT4 binds Mi2α and its close relative Mi2β, but not the other components of the repressive Mi2-containing NuRD complex. In the search for the biological role of this interaction, we find that PRMT4 and Mi2α/β interact with the transcription factor c-Myb and cooperatively coactivate c-Myb target gene expression in haematopoietic cell lines. This coactivation requires the methyltransferase and ATPase activity of PRMT4 and Mi2, respectively. Chromatin immunoprecipitation analysis shows that c-Myb target genes are direct transcriptional targets of PRMT4 and Mi2. Knockdown of PRMT4 or Mi2α/β in haematopoietic cells of the erythroid lineage results in diminished transcriptional induction of c-Myb target genes, attenuated cell growth and survival, and deregulated differentiation resembling the effects caused by c-Myb depletion. These findings reveal an important and so far unknown connection between PRMT4 and the chromatin remodeller Mi2 in c-Myb signalling.

## Author Summary

Our manuscript deals with the Protein arginine methyltransferase 4 (PRMT4), which modifies arginine residues in histones and other chromatin-associated proteins and plays an important role in the regulation of gene expression. We addressed the question of how the transcriptional function of PRMT4 might contribute to cell lineage specification despite its ubiquitious expression pattern and how this could explain its involvement in tumorigenesis. As protein associations are likely to provide an answer to this question, we attempted to identify novel interaction partners of PRMT4 using a biochemical approach. By this means, we found that PRMT4 binds Mi2α and its close relative Mi2β. In the search for the biological role of this interaction, we found that PRMT4 and Mi2α/β interact with the transcription factor c-Myb and cooperatively coactivate c-Myb target gene expression in haematopoietic cell lines. Depletion of PRMT4 or Mi2α/β in human erythroleukemia cells resulted in deregulated cell proliferation and differentiation resembling the effects caused by c-Myb depletion. Our findings unravel an important and so far unknown connection between PRMT4 and the chromatin remodeller Mi2 in c-Myb signalling and gene activation and identify both coregulators as attractive targets for leukaemia research and therapy in the future.

## Introduction

Protein arginine methyltransferases (PRMTs) constitute a family of nine members (PRMT1-9) in mammals, which are characterised by a conserved catalytic domain [1], [2]. They post-translationally mono- and dimethylate arginine residues in proteins using S-adenosylmethionine (SAM) as methyl group donor. Dimethylation can be either asymmetric or symmetric [3]. PRMTs regulate a plethora of cellular functions, including signal transduction, ribosome biogenesis, RNA processing, nucleo-cytoplasmic transport and chromatin-dependent processes, such as DNA repair, imprinting and transcriptional regulation, for which they usually require their catalytic activity. In agreement with their chromatin-related functions, a subgroup of PRMTs methylates histones as well as other chromatin-associated proteins and in this way contributes either to activation or repression of gene expression [4].

PRMT4, also named CARM1 (coactivator associated arginine methyltransferase 1), was the first member linked to transcriptional activation through asymmetric dimethylation of histone H3 at arginine 17 (H3R17me2a) [5]–[7]. Together with other coactivators, such as PRMT1 and the histone acetyltransferase (HAT) CBP/p300, PRMT4 is recruited to specific target genes through interaction with transcription factors, for example p53, NF-κB and nuclear hormone receptors such as the estrogen receptor (ER) [8]–[11]. The hierarchy of sequential coactivator recruitment in ER signalling has been studied in detail revealing that PRMT1-mediated dimethylation of histone H4 at arginine 3 (H4R3me2a) occurs as an early event following hormone treatment and is a prerequisite for promoter hyperacetylation [12], [13]. Subsequent histone acetylation by CBP/p300 facilitates promoter recognition by PRMT4 and methylation of H3R17 [14]. These various histone modifications at promoter-proximal nucleosomes of the target genes coincide with transcriptional activation.

PRMT1- and CBP/p300-mediated histone modifications are required for the subsequent recruitment and enhanced activity of coactivators explaining their direct support of active transcription [12], [14], [15]. Furthermore, histone acetylation is read by Bromo domain-containing proteins, such as the TAFII250 subunit of the TFIID complex, linking this modification directly to pre-initiation complex formation [16]. In the case of PRMT4 and H3R17 methylation, the mechanistic contribution is less clear, although the general relevance of PRMT4 and its catalytic activity in ER-dependent gene activation and embryonic development have been demonstrated in knockout and knockin mice [17], [18]. The recent identification of the Tudor domain-containing protein TDRD3 and the transcription elongation-associated PAF1 complex as readers of methylated H3R17 in the context of ER signalling provides a first hint to how this modification might directly promote transcriptional activation [19], [20]. Besides histone arginine methylation, modification of non-histone proteins plays a similarly important role for the transcriptional function of PRMT4. PRMT4 methylates HATs, such as CBP/p300 and SRC-3, thereby influencing their half-life and capability to interact with other proteins and thus modulating their coactivating function [21]–[24].

Similar to the majority of chromatin modifiers and transcriptional coregulators, PRMT4 seems to exert its functions not as an individual protein, but in close association with interaction partners or within multi-protein complexes. For example, PRMT4 was found in a complex of at least ten proteins, called the nucleosomal methylation coactivator complex (NUMAC), which includes components of the SWI/SNF remodeller complex and coactivates ER-dependent transcription in breast cancer cells [25]. As part of NUMAC PRMT4 acquires the ability to methylate nucleosomal histone H3, whereas recombinant PRMT4 preferentially methylates free H3. Such protein associations are likely to explain how PRMT4 contributes to cell type-specific functions and to cell lineage specification despite its ubiquitious expression pattern [5]. In the early embryo, PRMT4 regulates the development of the inner cell mass and activates expression of pluripotency markers [26], [27], whereas in differentiating skeletal muscle cells PRMT4 is required for the late myogenic transcription programme [28]. Deregulated expression of PRMT4 in certain tissues leads to aberrant transcription and is linked to tumorigenesis, such as high-grade breast tumors [29].

Further knowledge on interaction partners of PRMT4 would be necessary to understand its cell type-specific functions and its contribution to pathogenesis. In an attempt to identify such novel interaction partners of PRMT4 using a biochemical approach, we discovered here the ATP-dependent chromatin remodellers Mi2α and Mi2β, also called CHD3 and CHD4 (chromodomain-helicase-DNA binding protein 3 and 4) respectively, as such candidates. We found that PRMT4 coactivates c-Myb-dependent gene expression together with Mi2α as well as Mi2β in a cooperative manner. PRMT4 and Mi2 simultaneously occupy c-Myb target gene promoters in a c-Myb-dependent fashion and are regulators of cell survival and differentiation of the haematopoietic lineage resembling the function of c-Myb.

## Results

### Identification of novel interaction partners of PRMT4

**Figure 1:**
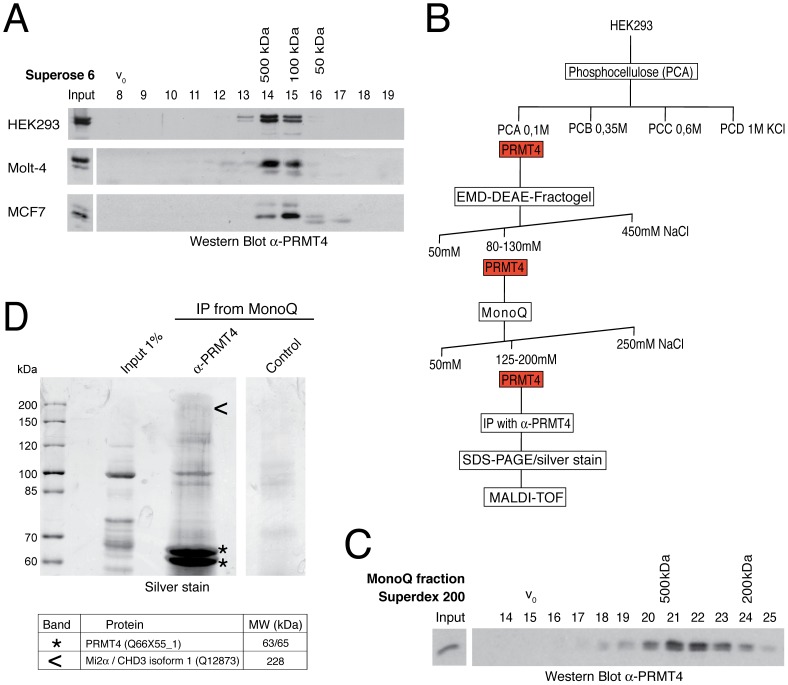
Identification of putative interaction partners of PRMT4. A: PRMT4 resides within a high molecular weight protein complex. For size fractionation by gel filtration chromatography, whole-cell protein extracts were generated from HEK293, Molt-4 and MCF7 cells and subjected to Benzonase treatment. Protein extracts were applied to a Superose 6 column and 1 ml fractions were collected. Fractions (8–19) were stained by Western Blot analysis using anti-PRMT4 antibodies. The column was calibrated using standard protein markers. Accordingly, the size of the PRMT4-containing fractions and the void volume (V_0_) are indicated. B: Biochemical purification of endogenous PRMT4. Schematic representation of the chromatographic steps used to purify PRMT4-containing complexes from HEK293 protein extract. C: High molecular weight complexes of PRMT4 remain stably associated during purification procedure. PRMT4-containing MonoQ fractions were size-fractionated using a Superdex 200 column. After collection of fractions (500 µl each), fraction numbers 14–25 were stained by Western Blot analysis using anti-PRMT4 antibodies. The size of the PRMT4-containing fractions and the void volume (V_0_) are indicated. D: Affinity purification of endogenous PRMT4 by immunoprecipitation. PRMT4-containing MonoQ fractions were subjected to IP using polyclonal anti-PRMT4 antibodies compared to bead control. Input (1% = 12 µg) and immunoprecipitates were separated by SDS-PAGE. Silver-stained bands specifically detected in the anti-PRMT4 sample were excised and identified by MALDI-TOF peptide mass fingerprint analysis as PRMT4 (asterisks) and Mi2α (arrowheads). SDS-PAGE size markers (in kDa) are shown on the left. Two bands were identified as PRMT4, which are likely different isoforms (of 63 and 65 kDa), but which could not be distinguished by peptides in the mass spectrometry.

To explore the molecular function of PRMT4 in gene regulation we aimed to identify novel interaction partners of the enzyme. We performed gel filtration analysis of protein extracts from several cell lines and detected PRMT4 by Western Blot analysis. In HEK293 and Molt-4 cells endogenous PRMT4 protein formed higher molecular weight complexes than expected from its monomeric or dimeric molecular weight (monomeric MW of PRMT4 = 65 kDa) and peaked in elution fractions of approximately 500 kDa (Figure 1A). Similar results were obtained for overexpressed PRMT4 in HEK293 cells (data not shown). In MCF-7 cells PRMT4 did not peak in the 500 kDa fraction, but significantly eluted with proteins of 100 kDa molecular weight (Figure 1A). These results indicate that PRMT4 stably associates with other proteins in higher molecular weight complexes in a cell type-dependent manner.

In order to purify endogenous PRMT4 with associated proteins we next performed cation and anion exchange chromatography of HEK293 extracts (Figure 1B). The presence of PRMT4 was detected by Western Blot analysis and by methyltransferase assay towards histone H3 in each chromatographic fraction (data not shown). PRMT4 did not bind the cation exchanger phosphocellulose, but eluted from the following anion exchangers DEAE and MonoQ at defined salt concentrations (Figure 1B). Thereby, PRMT4 was separated for example from the PRMT1 enzyme, which eluted from the DEAE column at a higher salt concentration (data not shown). To confirm that the high molecular weight complexes of PRMT4 remained stably associated during the ion exchange chromatography we performed gel filtration analysis after each chromatographic step and detected the presence of PRMT4 by Western Blot analysis, as exemplarily shown for the elution fractions of the MonoQ column (Figure 1C). Using the PRMT4-containing MonoQ fractions we performed affinity purification of the endogenous PRMT4 protein by immunoprecipitation (IP). Both anti-PRMT4 and control IPs were analysed by SDS-PAGE and silver staining (Figure 1D). Silver-stained protein bands specifically detected in the anti-PRMT4 samples were excised and protein identity was determined by mass spectrometry analysis. Among other proteins (not shown), we identified PRMT4 itself and the ATP-dependent chromatin remodeller Mi2α, also called CHD3 (chromodomain-helicase-DNA binding protein 3) (Figure 1D). Components of the NUMAC complex were not identified [25]. This result suggests that Mi2α is a putative interaction partner of PRMT4.

### Mi2α and Mi2β are novel interaction partners of PRMT4

**Figure 2:**
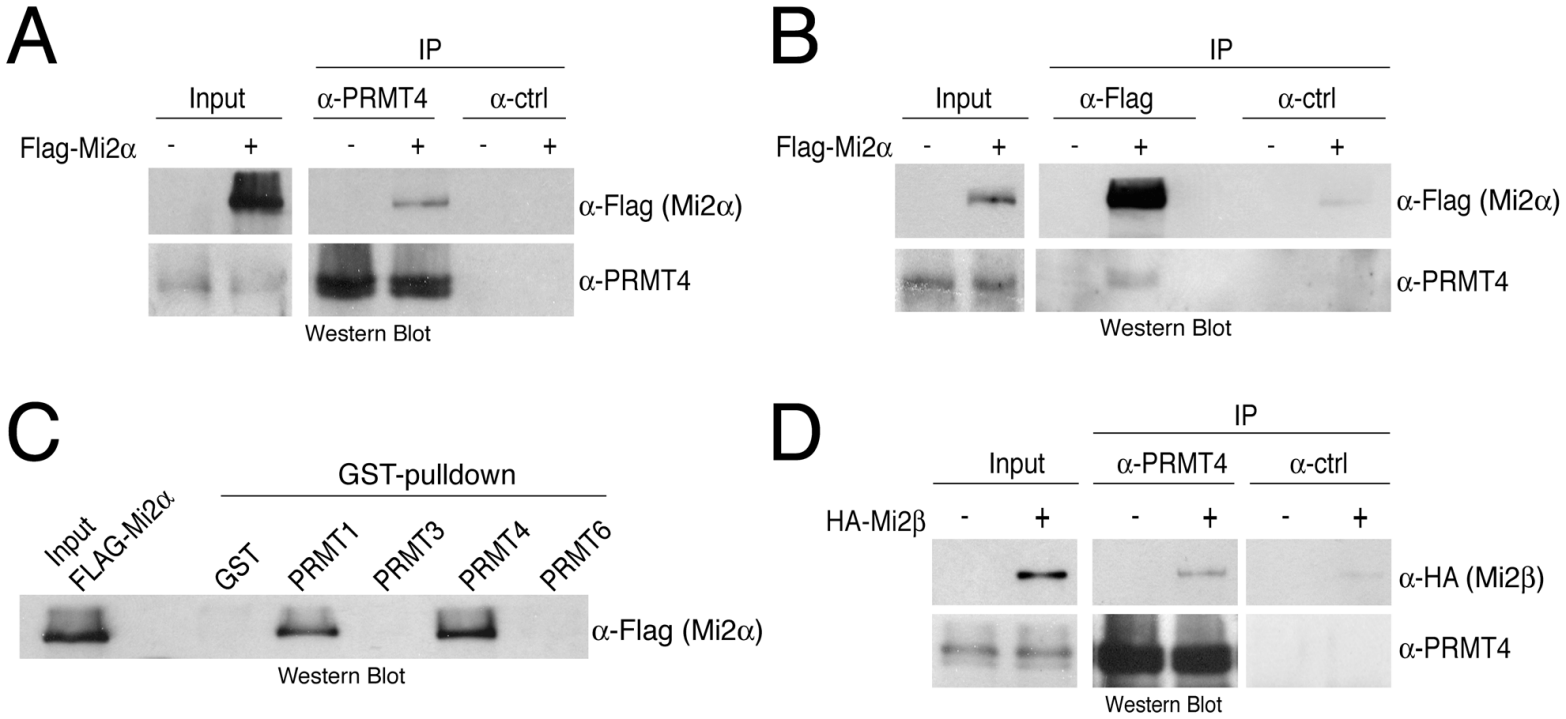
PRMT4 interacts with Mi2α and Mi2β. A, B: Co-immunoprecipitation of PRMT4 with Mi2α. HEK293 cells were transfected with Flag-Mi2α construct (+) or empty vector (−). After 48 hours, cells were lysed and 1 mg protein extract was subjected to IP of (A) endogenous PRMT4 (α-PRMT4) or (B) overexpressed Flag-Mi2α (α-Flag). IPs using isotype control IgG were performed in parallel (α-ctrl). Input (1%) and immunoprecipitates were stained by Western Blot analysis using anti-Flag and anti-PRMT4 antibodies. C: Among the PRMT family, PRMT4 preferentially interacts with Mi2α. Protein lysates of Flag-Mi2α-overexpressing HEK293 cells (as in A, B) were subjected to GST-pulldown experiments. Equal amounts of recombinant proteins GST alone and GST-PRMT1, -3, -4 and -6 (Figure S1) coupled to glutathione beads were incubated with 250 µg of HEK293 extracts. Input (1%) and bound Flag-Mi2α protein were visualised by Western Blot analysis using anti-Flag antibodies. D: Co-immunoprecipitation of PRMT4 with Mi2β. HEK293 cells were transfected with HA-Mi2β construct (+) or empty vector (−). Protein extracts were subjected to IP using anti-PRMT4 (α-PRMT4) antibodies or isotype control IgG (α-ctrl). Input (1%) and precipitates were stained by Western Blot analysis using anti-HA and anti-PRMT4 antibodies.

We next analysed the putative interaction between PRMT4 and Mi2α by performing co-immunoprecipitations (co-IP) followed by Western Blot analysis. Immunoprecipitation (IP) of endogenous PRMT4 from HEK293 cells copurified overexpressed Flag-tagged Mi2α (Figure 2A) and reciprocally immunoprecipitates of Flag-Mi2α revealed the presence of endogenous PRMT4 (Figure 2B). IP of Mi2α from the PRMT4-enriched MonoQ fractions using a newly generated anti-Mi2α serum revealed also an interaction between PRMT4 and Mi2α on the endogenous level and supported the mass spectrometrical result (Figure S1). To address the question of whether other PRMTs, such as PRMT1, PRMT3 and PRMT6, are also able to interact with Mi2α we performed GST-pulldown assays. Flag-Mi2α preferentially bound to the GST-fusion of PRMT4 and to a lower extent to GST-PRMT1, but not to the other tested PRMT members (Figure 2C, Figure S2). These results confirm the specificity of this novel interaction and identify PRMT4 as the predominant PRMT to interact with Mi2α.

Given that Mi2α has a close relative, Mi2β/CHD4, we asked whether Mi2β could interact as well with PRMT4. Co-IP analysis revealed an interaction between HA-tagged Mi2β and PRMT4 (Figure 2D). Both Mi2 proteins harbour several conserved functional domains [30], including two PHD fingers, which possess individual histone-binding activities enabling bivalent recognition of two histone H3 tails within nucleosomes [31], [32], two Chromo domains that bind DNA [33] and a SNF2-type ATPase domain. To map the interaction domain of PRMT4 in Mi2 we expressed and radiolabeled Mi2α deletion mutants in an IVT system and performed pulldown experiments with GST-PRMT4. The deletion constructs contained either the N-terminus, the two PHD domains, the two Chromo domains, the helicase domain or the C-terminus (Figure S3). This assay revealed that PRMT4 interacts with the N-terminal region and the Chromo domains of Mi2α (Figure S3). Together, these results show that PRMT4 interacts with both Mi2 proteins and narrow down, as exemplified for Mi2α, the interaction surface of PRMT4 in Mi2.

Mi2α and Mi2β have been reported to be part of the NuRD (nucleosome remodelling and deacetylation) complex and accordingly to function in transcriptional repression, as NuRD provides a physical link between ATP-dependent chromatin remodelling and HDAC (histone deacetylase) activity [34]–[36]. Therefore we investigated whether PRMT4 associates with other subunits of the NuRD complex. In co-IP assays we confirmed the interaction of Mi2α with the NuRD components MBD3 or HDAC1 (Figure S4). However, specific interactions with MBD3 or HDAC1 were not detected in the PRMT4 immunopreciptates. These results indicate that PRMT4 selectively binds both Mi2 proteins, but no other components of the NuRD complex suggesting that the PRMT4-Mi2 interaction might not be linked to NuRD-mediated transcriptional repression.

### PRMT4 and Mi2 interact with the transcription factor c-Myb

**Figure 3:**
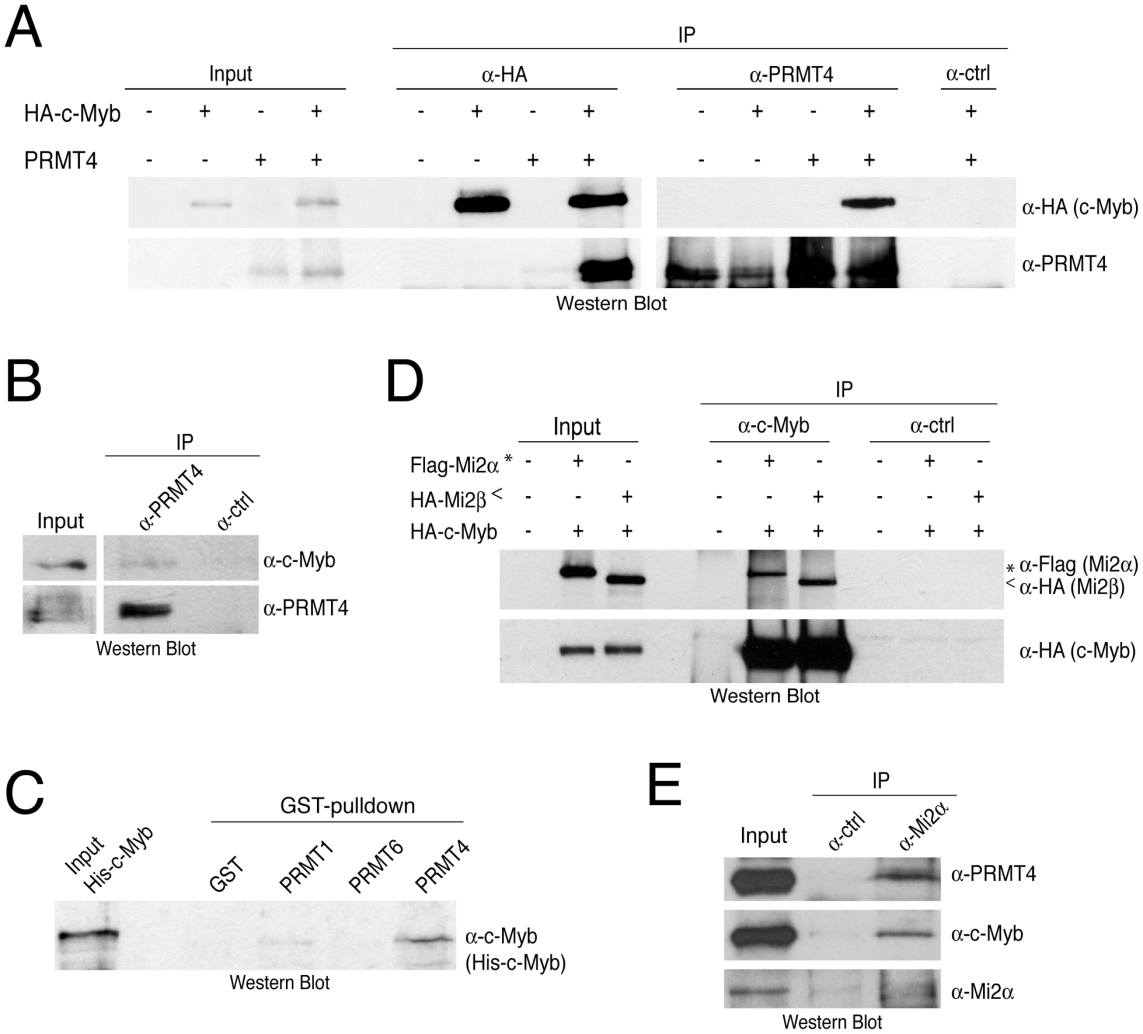
PRMT4 and Mi2 interact with the transcription factor c-Myb. A: Co-immunoprecipitation of overexpressed PRMT4 with c-Myb. HEK293 cells were transfected with untagged PRMT4 and HA-c-Myb construct (alone or in combination) and harvested 48 hours after transfection. Protein extracts were subjected to IP using anti-HA (α-HA), anti-PRMT4 (α-PRMT4) antibodies or isotype control IgG (α-ctrl). Input (1%) and precipitates were stained by Western Blot analysis using anti-HA and anti-PRMT4 antibodies. B: Co-immunoprecipitation of endogenous PRMT4 with c-Myb. Jurkat cell extract was incubated with anti-PRMT4 (α-PRMT4) or isotype control IgG (α-ctrl). Input (5%) and precipitates were stained by Western Blot analysis using anti-c-Myb and anti-PRMT4 antibodies. The anti-c-Myb stainings for input and IP shown in the panels derive from the same blot and exposure time revealing that less than 5% of endogenous c-Myb interacts with endogenous PRMT4. C: PRMT4 and c-Myb are direct interaction partners. GST-pulldown with recombinant His-tagged c-Myb was performed using equal amounts of glutathione beads-bound recombinant GST, GST-PRMT1, -4 and -6 proteins. Input and bound His-c-Myb were visualised by Western Blot analysis using anti-c-Myb antibodies. D: Mi2α and Mi2β interact with c-Myb. HEK293 cells were transfected with HA-c-Myb plasmid together with Flag-Mi2α or HA-Mi2β. Protein extracts were incubated with anti-c-Myb antibodies or isotype control IgG (α-ctrl). Input and precipitates were stained by Western Blot analysis using anti-HA and anti-Flag antibodies. Asterisks indicate Flag-Mi2α and arrowheads indicate HA-Mi2β. E: Co-immunoprecipitation of endogenous Mi2α with PRMT4 and c-Myb. Jurkat cell extract was incubated with anti-Mi2α (α-Mi2α) or control IgG (α-ctrl). Input (5%) and precipitates were stained by Western Blot analysis using anti-PRMT4, anti-c-Myb and anti-Mi2α antibodies.

Apart from their repressive function within the NuRD complex, both Mi2 proteins are also involved in transcriptional activation. For example, human Mi2β is required for T cell development and activation of the *CD4* gene [37]. The Drosophila orthologue of Mi2β/CHD4 is localised to actively transcribed regions of polytene chromosomes [38]. Furthermore, Mi2α coactivates c-Myb-mediated transcription independently of its helicase activity [39]. The proto-oncogenic transcription factor c-Myb plays a central role in the proliferation and differentiation of different haematopoitic lineages, in particular of erythrocytes and thymocytes [40], [41], and similarly PRMT4 and Mi2 knockout studies revealed severe defects in early T-cell development [37], [42]. This led us to investigate whether PRMT4 as well as both Mi2 proteins are able to interact with c-Myb. HEK293 cells were transfected with untagged PRMT4 and HA-tagged c-Myb and co-IP assays were performed with antibodies against PRMT4, HA or IgG control. We detected PRMT4 in HA-c-Myb-immunoprecipitates and reciprocally HA-tagged c-Myb in PRMT4-immunoprecipitates (Figure 3A). Both proteins also interacted endogenously in the T lymphocyte cell line Jurkat (Figure 3B). Moreover, pulldown assays using GST-PRMTs and bacterially expressed His-tagged c-Myb revealed a preferential and direct interaction between c-Myb and GST-PRMT4, whereas GST-PRMT1 exhibited a weak interaction and GST-PRMT6 no interaction with c-Myb (Figure 3C). Furthermore, we showed that Flag-tagged Mi2α and HA-tagged c-Myb coimmunoprecipitate (Figure 3D, Figure S5), as previously reported [39]. Additionally, using the same approach we uncovered that also Mi2β was able to interact with c-Myb (Figure 3D). Using protein extracts from Jurkat cells, which reveal high expression levels of PRMT4, c-Myb and Mi2 (data not shown), we validated that Mi2α interacts with c-Myb and PRMT4 also endogenously (Figure 3E). These results identify PRMT4 and both Mi2 proteins as novel interaction partners of the c-Myb transcription factor.

### PRMT4 and Mi2 cooperate in the coactivation of c-Myb-dependent transcription

**Figure 4:**
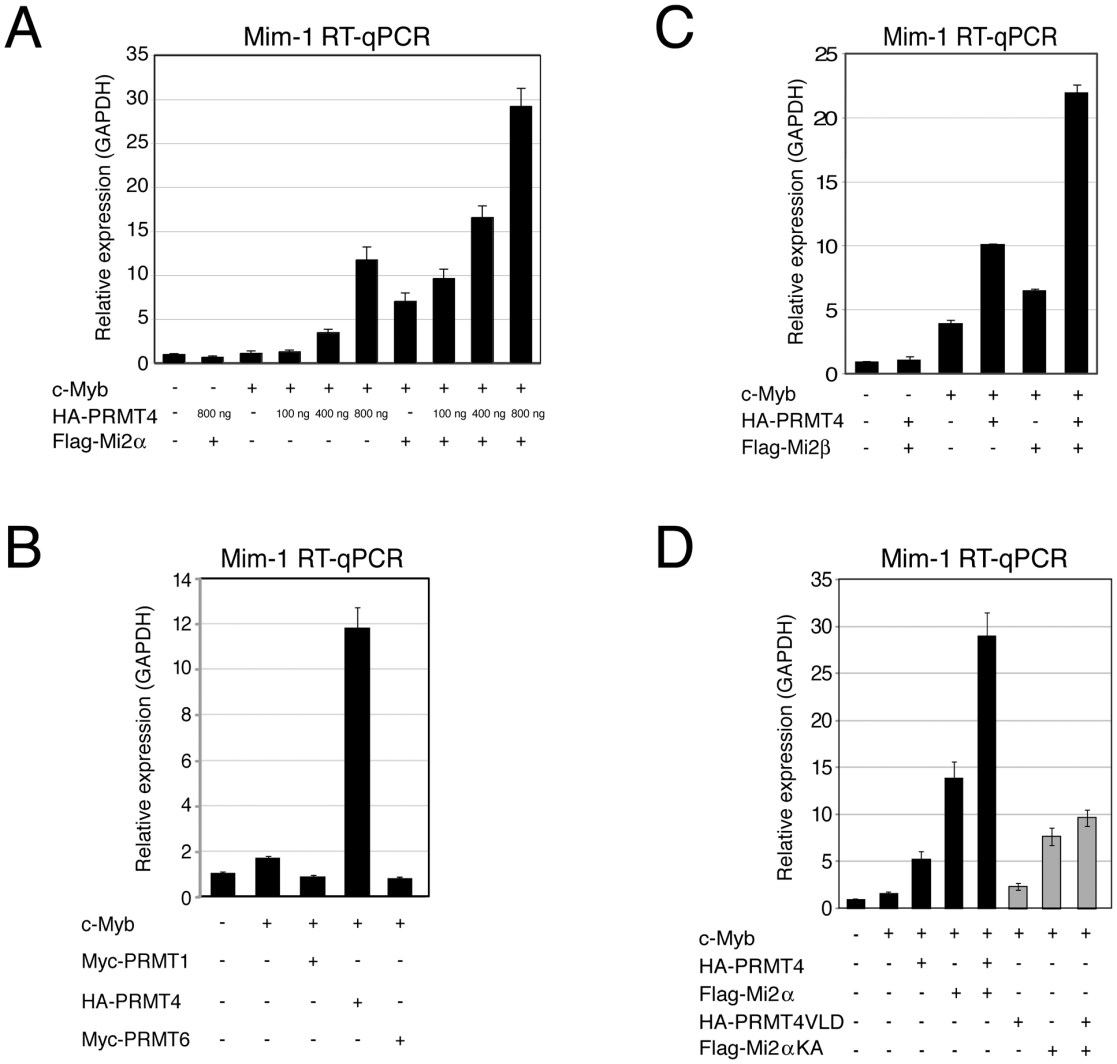
PRMT4 and Mi2 are cooperating transcriptional activators of c-Myb-dependent gene expression in the chicken macrophage cell line HD11. A: PRMT4 and Mi2α are synergistic coactivators of c-Myb target genes. HD11 cells were transfected with the indicated constructs. After 48 hours, cells were harvested and total RNA was isolated. RT-qPCR was performed for detection of transcript levels of *Mim-1*. Each mRNA expression was normalised to *GAPDH* mRNA expression. Transcript levels in empty vector-transfected cells (−) were set to 1. B: Coactivation of c-Myb-dependent gene expression is specific for PRMT4. HD11 cells were transfected with the indicated constructs. After 48 hours, cells were harvested for total RNA isolation. Levels of *Mim-1* mRNA were analysed by RT-qPCR and normalised to *GAPDH* mRNA levels. Transcript levels in empty vector-transfected cells (−) were set to 1. C: Mi2β synergises with PRMT4 in c-Myb-dependent gene expression. HD11 cells were transfected with indicated constructs. After 48 hours, cells were harvested for total RNA isolation. Levels of *Mim-1* mRNA were analysed by RT-qPCR and normalised to *GAPDH* mRNA levels. Transcript levels in empty vector-transfected cells (−) were set to 1. D: The catalytic activity of PRMT4 and Mi2 is essential for their cooperative function. HD11 cells were transfected with tagged wild type (black) and catalytic mutant forms (grey) of PRMT4 and Mi2α (methyltransferase-dead PRMT4 mutant: VLD; helicase-dead Mi2α mutant: KA). Total RNA was isolated 48 hours after transfection. Levels of *Mim-1* mRNA were analysed by RT-qPCR and normalised to *GAPDH* mRNA levels. Transcript levels of empty vector-transfected cells (−) were set to 1.

To address whether PRMT4 together with Mi2α and Mi2β regulates the transcriptional activity of c-Myb, we employed the chicken myelomonocytic cell line HD11 that does not endogenously express c-Myb, but is competent to induce endogenous c-Myb target genes, such as *Mim-1* and *Lysozyme*, upon overexpression of c-Myb [43], [44]. *Mim-1* is one of the best-characterised Myb target genes and its transcription is strongly upregulated by c-Myb designating the gene an excellent model to study the influence of transcriptional coregulators [45]. We transfected HD11 cells with c-Myb alone or in combination with PRMT4 and Mi2, which both did not affect the expression levels of c-Myb itself (Figure S6), and measured the levels of *Mim-1* and *Lysozyme* transcripts by reverse transcription-quantitative PCR (RT-qPCR). We found that increasing amounts of PRMT4 enhanced the transcript levels of *Mim-1* and *Lysozyme* in a c-Myb-dependent and concentration-dependent manner (Figure 4A, Figure S7). In contrast, overexpression of PRMT1 and PRMT6 did not augment the transcriptional activity of c-Myb, as exemplified for the *Mim-1* and *Lysozyme* gene (Figure 4B, Figure S8), rather PRMT6 repressed the c-Myb-mediated activation in line with its corepressor function [46], [47]. Noticeably, coexpression of PRMT4 and Mi2α further enhanced the transcriptional activity of c-Myb (Figure 4A, Figure S7). The same result was obtained for coexpression of PRMT4 and Mi2β (Figure 4C) suggesting that PRMT4 cooperates with Mi2α and Mi2β in coactivating c-Myb target gene transcription. This effect required the catalytic activity of PRMT4, as overexpression of a methyltransferase-deficient mutant of PRMT4 (VLD) resulted in the loss of coactivation of c-Myb (Figure 4D, Figure S9). Similarly, overexpression of an ATPase-deficient mutant of Mi2α (KA) led to a reduced coactivation in case of *Mim-1* and to a loss of coactivation in case of *Lysozyme* gene expression (Figure 4D, Figure S9). Moreover, for both target genes the cooperativity between PRMT4 and Mi2 was impaired upon transfection of both catalytic mutants. Together, these data indicate that PRMT4 is a novel coactivator of the c-Myb transcription factor and synergises with both Mi2 proteins in a methyltransferase- and helicase-dependent manner to coregulate c-Myb activity.

**Figure 5:**
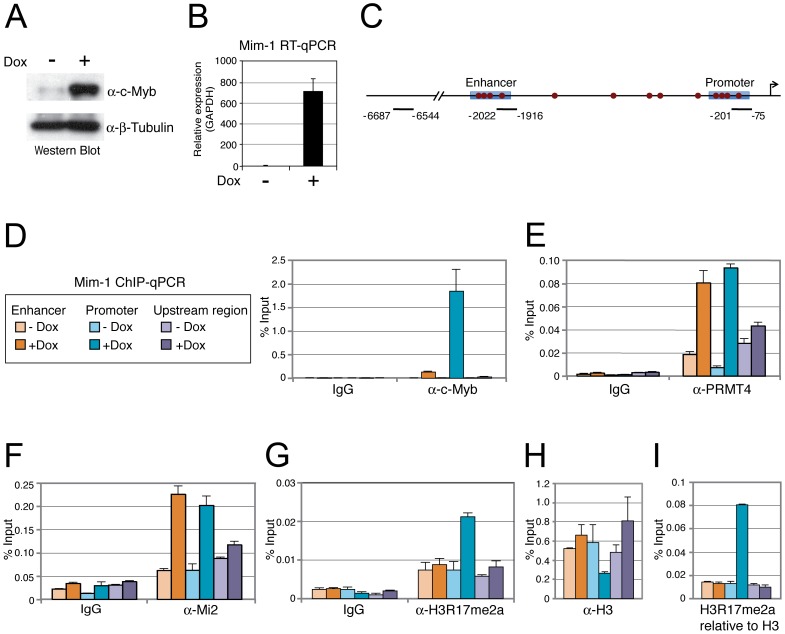
PRMT4 and Mi2 are concomitantly recruited with c-Myb to the *Mim-1* gene. A, B: HD11-C3 cells stably express a doxycycline-inducible c-Myb construct. HD11-C3 cells were either left untreated (−Dox) or treated with doxycycline (+Dox) for 30 hours. For isolation of protein extracts and total RNA cells were harvested. (A) Levels of c-Myb protein were detected by Western Blot analysis and β-Tubulin staining served as a loading control. (B) Transcript levels of *Mim-1* were measured by RT-qPCR and normalised to *GAPDH* mRNA levels. The mRNA level in untreated cells (−) was set to 1. C: Schematic representation of the *Mim-1* gene upstream of the transcriptional start site (indicated by arrow). Enhancer and promoter regions (blue rectangles) and the c-Myb binding site (red dots) are marked. The amplicon of the promoter region (−201 to −75), enhancer region (−2022 to −1916) and upstream control region (−6687 to −6544) generated in the ChIP-qPCR (D) are indicated. D–H: PRMT4 and Mi2 are direct transcriptional regulators of c-Myb target gene *Mim-1*. HD11-C3 cells were treated as in A, B and harvested for chromatin isolation. Chromatin was subjected to ChIP analysis using antibodies against c-Myb (D), PRMT4 (E), Mi2 (F), H3R17me2 (G, I), histone H3 (H, I) and control antibody (IgG). Immunoprecipitated DNA was analysed in triplicates by qPCR with primers spanning the regions of the *Mim-1* gene indicated in C (orange = enhancer; blue = promoter; purple = upstream control region). Legend in D also applies to E–I. Mean values were expressed in % input of chromatin.

To analyse whether the effect of PRMT4 and Mi2 on *Mim-1* gene activation correlates with their concomitant recruitment to the *Mim-1* regulatory regions, in which c-Myb binding sites have been identified [43]–[45], [48], we performed chromatin immunoprecipitation (ChIP). We used HD11 cells stably expressing a doxycycline-inducible c-Myb construct (HD11-C3). In response to doxycycline, the levels of c-Myb protein (Figure 5A) and consistently of *Mim-1* transcript (Figure 5B) were increased. ChIP analysis revealed that upon doxycycline treatment c-Myb binds the promoter and to a lower extent the enhancer of the *Mim-1* gene, whereas an upstream control region was not occupied by c-Myb (Figure 5C, 5D). Recruitment of both PRMT4 and Mi2 was detected at the *Mim-1* promoter as well as enhancer in a c-Myb dependent manner, but not at the upstream control region (Figure 5E, 5F). Therefore the recruitment of the two coactivators did not reflect the binding preference of c-Myb for the promoter. The occurrence of H3R17 methylation (H3R17me2a) exclusively correlated with the binding of PRMT4 at the promoter of the *Mim-1* gene (Figure 5G), suggesting that histone H3 is the substrate of PRMT4 preferentially at the promoter but not at the enhancer. Concomitantly with transcriptional induction of *Mim-1*, a reduction of histone H3 occupancy was detectable at the promoter (Figure 5H). When this decrease in total H3 levels was taken into account for the calculation of H3R17 methylation, its promoter-specific increase was even enhanced (Figure 5I). These findings indicate that the c-Myb-dependent *Mim-1* gene is a direct target of PRMT4 and Mi2 and that the two coactivators are concomitantly recruited with c-Myb.

### PRMT4 and Mi2 regulate c-Myb-dependent gene expression in K562 cells

**Figure 6:**
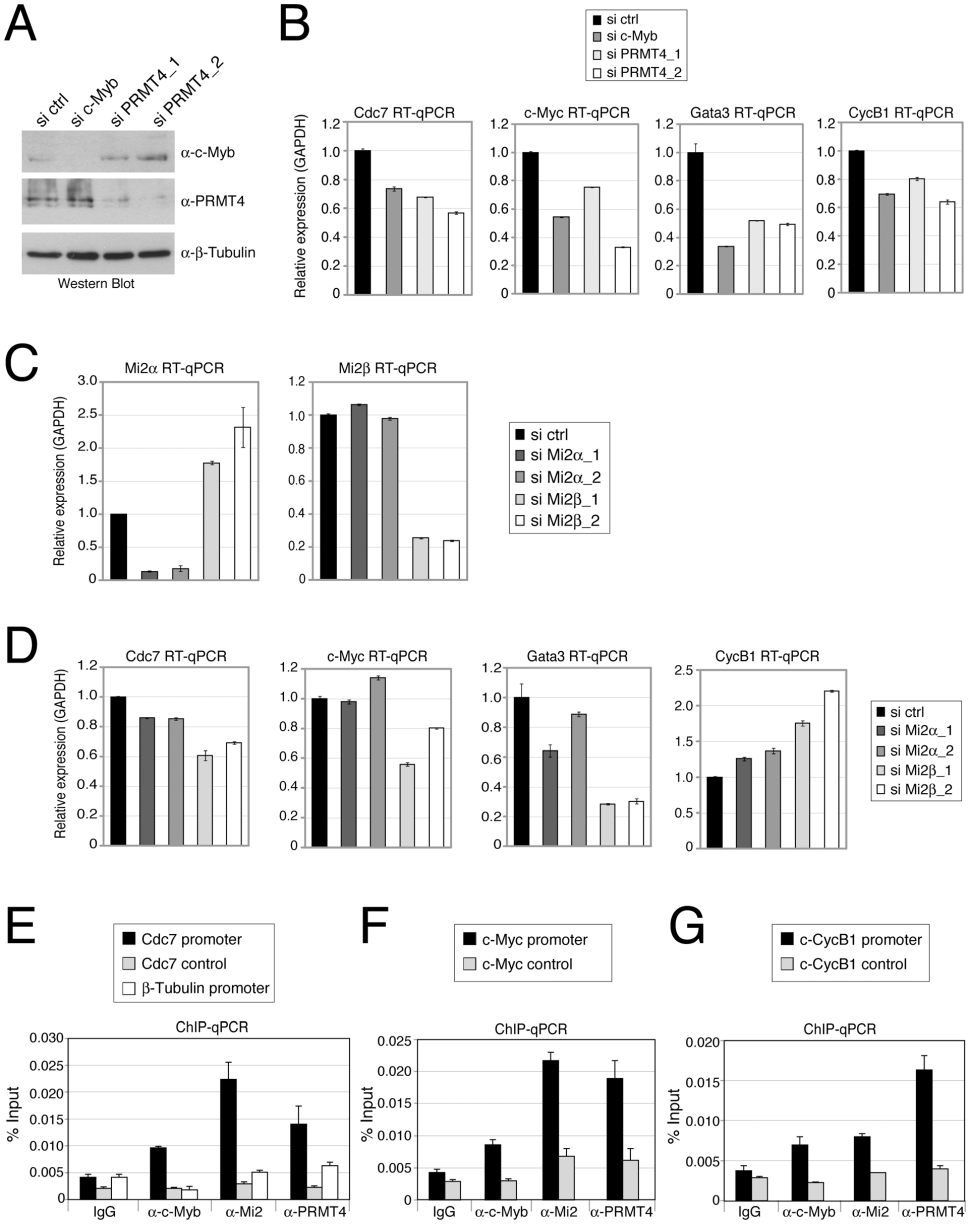
PRMT4 and Mi2 are direct transcriptional regulators of c-Myb target genes in human haematopoietic cells. A–D: PRMT4 and Mi2 regulate overlapping c-Myb target genes in K562 cells. Knockdown of c-Myb, PRMT4, Mi2α and Mi2β was achieved in K562 by electroporation of the corresponding siRNAs. Cells were harvested 3 days later and protein extracts and total RNA were prepared. (A) For estimation of knockdown efficiency, protein levels of c-Myb, PRMT4 and as loading control β-Tubulin were measured by Western Blot analysis. (C) For detection of knockdown efficiency of *Mi2α* and *Mi2β* at mRNA levels, RT-qPCR was conducted using gene-specific primers for *Mi2α* and *Mi2β* and normalised to *GAPDH*. Transcript levels in si ctrl-treated cells were set to 1. (B, D) Transcript levels of the c-Myb target genes *Cdc7*, *c-Myc*, *Gata3* and *CyclinB1 (CycB1)* were measured in the various knockdown conditions by RT-qPCR and normalised to *GAPDH*. Transcript levels in si ctrl-treated cells were set to 1. E–G: Concomitant recruitment of c-Myb, PRMT4 and Mi2 to c-Myb target genes in human haematopoietic cells. K562 cells were harvested and subjected to ChIP analysis using control antibodies (IgG) and antibodies against c-Myb, PRMT4 and Mi2. Immunoprecipitated DNA was analysed by qPCR with primers for (E) *Cdc7* promoter, corresponding control region and *β-Tubulin* promoter, for (F) *c-Myc* promoter and corresponding control region and for (G) *Cyclin B1 (CycB1)* promoter and corresponding control region. Recruitment is displayed in % input of chromatin.

We next investigated whether PRMT4 and Mi2 were also relevant transcriptional coactivators of c-Myb in mammalian cells. Given that c-Myb is a key regulator of haematopoiesis, we decided to use the CML-derived erythroleukemic cell line K562, in which numerous c-Myb target genes have been identified [49], and performed siRNA-mediated depletion of c-Myb, PRMT4, Mi2α or Mi2β. For each knockdown condition, we then analysed the mRNA levels of published c-Myb targets. Depletion of c-Myb, as documented by Western Blot analysis (Figure 6A), led to a decrease in transcript levels of *Cdc7*, *c-Myc*, *Gata3* and *CycB1* (Figure 6B) as previously reported [49], [50]. Noticeably, the transcript levels of these genes were reduced to the same extent after PRMT4 depletion (Figure 6A, 6B). These results indicate that an overlapping set of target genes is regulated by c-Myb and PRMT4 in human haematopoietic cells.

Next we asked whether expression of the same c-Myb target genes is influenced by Mi2α and Mi2β. K562 cells efficiently depleted of either Mi2α or Mi2β subsequent to siRNA transfection (Figure 6C) exhibited in most cases reduced transcript levels of the above identified PRMT4-regulated c-Myb targets (Figure 6D). The mRNA levels of *Cdc7*, *c-Myc* and *Gata3* were downregulated, most strongly upon Mi2β depletion, whereas knockdown of Mi2α showed a weaker (*Cdc7*, *Gata3*) or no effect (*c-Myc*, Figure 6D). In contrast, transcript levels of *CycB1* were up-regulated upon Mi2 depletion, again most strongly upon Mi2β depletion, suggesting that both proteins exert a repressive function in this case. Comparison of the mRNA levels of both Mi2 in wild type K562 cells revealed that Mi2β is predominantly expressed (data not shown), which might explain its stronger effects on gene expression in these cells. Together, these results reveal that PRMT4 and Mi2 influence an overlapping set of c-Myb target genes in human haematopoietic cells. All tested c-Myb targets were activated by PRMT4, whereas Mi2 operated in a gene-specific manner either as an activator or repressor. In case of *Cdc7*, *c-Myc* and *Gata3* genes, PRMT4 as well as Mi2 enhanced transcription corroborating our findings on a synergism of the two coregulators in c-Myb signalling.

In order to study whether PRMT4 and Mi2 proteins bind the regulatory regions of these c-Myb target genes, we performed ChIP analysis in K562 cells. We found that the promoter regions of *Cdc7*, *c-Myc* and *CycB1* genes, that contain c-Myb binding sites, were enriched in the immunoprecipitates of c-Myb, PRMT4 as well as Mi2 compared to the IgG control (Figure 6E–6G). In contrast, control regions of the three genes and the *β-Tubulin* gene promoter, which are free of c-Myb binding sites, were not bound by c-Myb or the two coregulators (Figure 6E–6G). These results indicate that PRMT4 and Mi2 are concomitantly recruited together with c-Myb to target gene promoters and are directly involved in coactivating a subset of c-Myb-dependent genes in human haematopoietic cells.

### PRMT4 and Mi2 are important regulators of proliferation and differentiation in K562 cells

The c-Myb target genes coregulated by PRMT4 and Mi2 in K562 cells fulfil well-established functions in cell cycle and proliferation control. Given that c-Myb is essential for the self-renewal and proliferative capacity of haematopoietic progenitor cells and suppresses differentiation [49], we next explored the biological significance of PRMT4 and Mi2 for the regulation of c-Myb activity in haematopoiesis. For this purpose we used K562 cells as a model for haematopoietic cell proliferation and differentiation.

**Figure 7:**
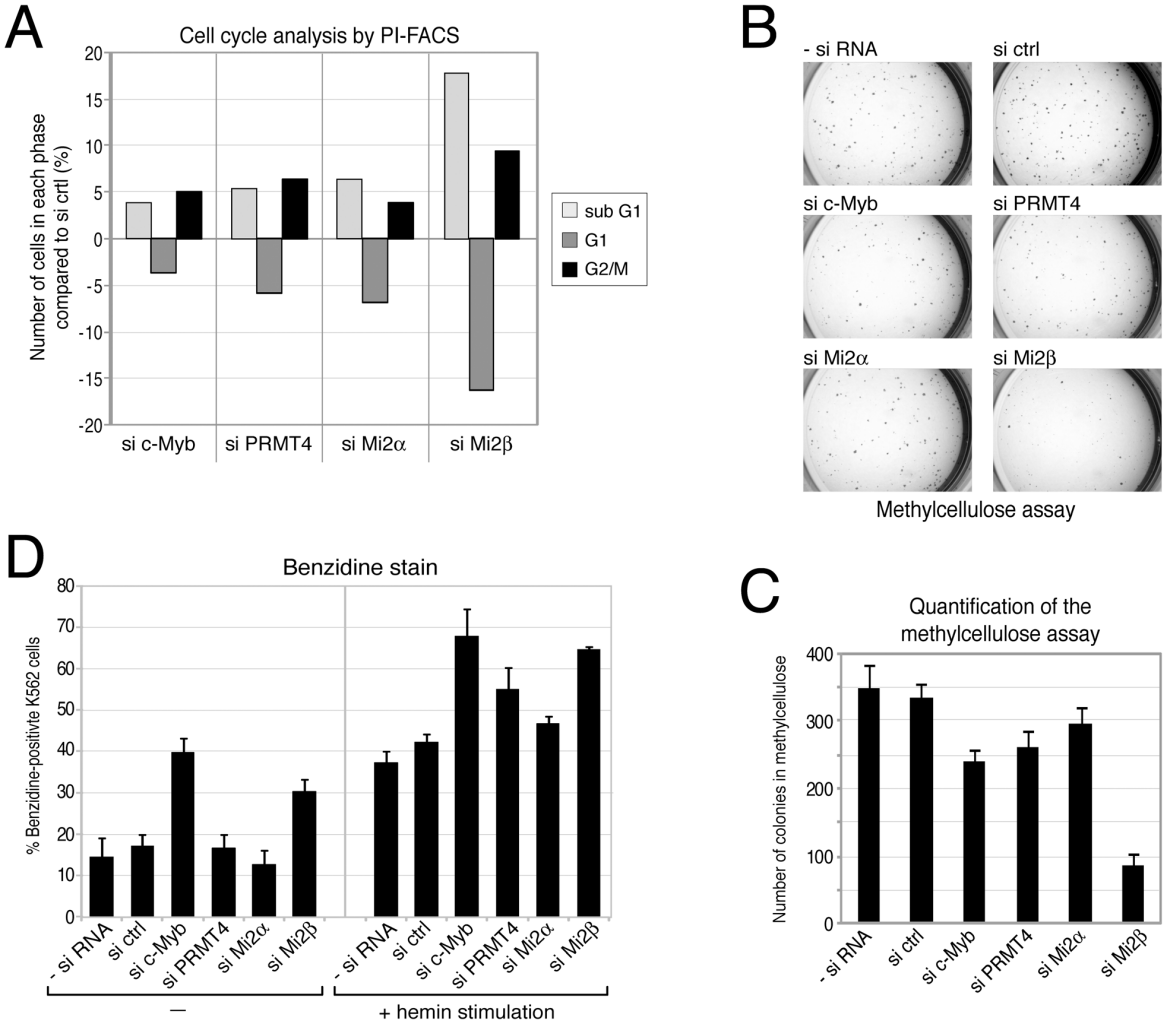
PRMT4 and Mi2 are important regulators of proliferation and differentiation function in human erythropoiesis. A: PRMT4 and Mi2 have similar effects on cell cycle progression to c-Myb. K562 cells were transfected with siRNAs targeting c-Myb, PRMT4, Mi2α and Mi2β or with control siRNA (si ctrl). The DNA content of propidium iodide (PI)-stained cells was measured by flow cytometry (FACS). From each knockdown the percentage of cells in sub-G1, G1 and G2/M phase was determined. Shown are the changes in percentage (%) relative to the si ctrl condition. A representative data set is depicted. B, C: PRMT4 and Mi2 have similar pro-proliferative functions to those of c-Myb. K562 cells were transfected with siRNAs, as in A. After 3 days of transfection, cells were cultured in methylcellulose for 2 days and stained with INT (Iodonitrotetrazolium chloride) for 5 days. (B) Representative pictures of the colonies were taken for each knockdown condition at the same magnification. (C) For quantification, colonies larger than 0.1 mm were counted. The mean of colony numbers was calculated from three independent experiments and error bars are indicated accordingly. D: Depletion of PRMT4 and Mi2 recapitulates the pro-differentiating effects of c-Myb knockdown. K562 cells were transfected with the indicated plasmids for 3 days. Cells were then re-seeded and either left untreated or treated with 30 µM hemin for 3 days. Subsequently, benzidine staining of differentiated cells was performed. The mean percentage (%) of benzidine-positive K562 cells was determined from triplicate countings and error bars are indicated accordingly.

First we intended to clarify the role of these novel coactivators in c-Myb-dependent proliferation. In agreement with earlier reports showing that inactivation of c-Myb in K562 cells leads to G2/M arrest [51], we found an increased cell number in the G2/M phase upon siRNA-mediated knockdown of c-Myb compared to control siRNA-transfected K562 cells, as analysed by propidium iodide (PI) FACS (Figure 7A, Figure S10). Furthermore, the number of cells in G1 phase was reduced, while the number of apoptotic cells (sub-G1 peak) was increased in c-Myb-depleted cells, corroborating the pro-proliferative and anti-apoptotic capacity of c-Myb in these cells. Next, we investigated whether depletion of PRMT4 and Mi2α/Mi2β, respectively, affects cell cycle distribution of K562 cells. Similarly, knockdown of the coactivators resulted in a G2/M arrest and in an increased apoptotic rate (Figure 7A, Figure S10). Depletion of Mi2β had a stronger effect on the cell cycle distribution than depletion of the other two coregulators or of c-Myb itself revealing that Mi2β might be implicated in additional c-Myb-independent pro-proliferative functions. To investigate the c-Myb-dependence of the PRMT4 as well as Mi2 function in cell cycle regulation, we depleted the three coactivators in U2OS cells, which clearly expressed lower levels of c-Myb compared to K562 cells, and monitored their cell cycle profile by PI-FACS (Figure S11). We found that PRMT4- or Mi2-depleted U2OS cells revealed no effect on apoptosis or at most a slightly decreased number of apoptotic cells in contrast to the enhanced apoptosis in K562 cells. However, depletion of the coactivators resulted in a decreased number of U2OS cells in G1 phase and in a G2/M arrest similar to our findings in PRMT4- and Mi2-depleted K562 cells suggesting that the apoptotic effects of PRMT4 and Mi2 might be c-Myb-dependent. These data show that PRMT4 and both Mi2 proteins regulate the cell cycle of K562 cells similar to c-Myb and might be functionally relevant coactivators of c-Myb with respect to its apoptotic function in haematopoietic cells.

Transformed cell lines are able to proliferate and form colonies in semi-solid medium. We then asked whether depletion of c-Myb or the coactivators influences this property of K562 cells. Depletion of either c-Myb, PRMT4, Mi2α or Mi2β resulted in reduced numbers of colonies in the methylcellulose colony formation assay compared to control transfected cells (Figure 7B, 7C). Again in this assay, the effect of Mi2β depletion exceeded the effect of c-Myb knockdown hinting at an additional c-Myb-independent role for Mi2β. These results independently confirm a pro-proliferative function of PRMT4 and Mi2 resembling the proliferation-promoting effect of c-Myb.

Finally, we aimed to investigate the potential function of the novel coactivators in the differentiation-suppressive activity of c-Myb. Expression levels of c-Myb are elevated in haematopoietic progenitors of different lineages including the myeloid lineage and decrease during differentiation into the various sub-lineages to allow cell cycle exit and terminal differentiation [49]. K562 cells maintain characteristics of multipotent haematopoietic progenitors and are able to differentiate along the erythroid lineage when treated with hemin [52]. Overexpression of c-Myb has been shown to specifically restrain the chemically induced differentiation of K562 along the erythroid lineage [53]. Therefore, we investigated whether depletion of c-Myb or its coregulators could enhance the hemin-induced differentiation of K562 cells. In the absence of hemin treatment, depletion of c-Myb as well as Mi2β enhanced the spontaneous differentiation into erythrocytes compared to the other siRNA-transfected cells, as quantified by staining of benzidine-positive cells (Figure 7D). Furthermore, the hemin-induced erythroid differentiation was enhanced upon depletion of c-Myb, PRMT4 and Mi2β. A weak increase was also obtained upon depletion of Mi2α (Figure 7D). Together with our findings that PRMT4 and Mi2 interact with c-Myb and coactivate c-Myb-dependent gene expression, these results suggest that PRMT4 and Mi2 are relevant coregulators of c-Myb and might contribute to its differentiation-blocking activity in human erythropoiesis.

## Discussion

In this study we searched for novel chromatin-associated interaction partners of the arginine methyltransferase PRMT4 to extend our understanding of its physiological and pathophysiological roles in transcriptional regulation. We found that PRMT4 forms stable complexes with other proteins as observed by gel filtration analysis, which revealed a cell type-specific elution profile of PRMT4. In HEK293 and Molt-4 extracts, PRMT4 is part of protein complexes that peak around 500 kDa, whereas MCF7 extracts predominantly showed PRMT4 in fractions of 100 kDa in size. Interestingly, the PRMT4-containing NUMAC complex was isolated from hormone-treated MCF7 cells using epitope-tagged PRMT4 [25] indicating that PRMT4 forms dynamic associations not only in a cell type- but also stimulus-dependent manner.

In search for novel interaction partners of PRMT4 we combined ion exchange chromatography of HEK293 protein extracts with endogenous co-IP of PRMT4 and mass spectrometrical analysis. The silver-stain analysis of the separated PRMT4 immunoprecipitates revealed that copurifying protein bands did not occur in stoichiometric ratio with PRMT4. Thus, the purification does not contain a predominant PRMT4-containing complex, rather PRMT4 temporarily and independently interacts with several proteins and these multiple interactions cause the molecular weight shift of PRMT4 in the gel filtration analysis. Components of the NUMAC complex were not identified in the mass spectrometrical analysis, which conforms to the fact that the PRMT4-containing complexes in HEK293 cells are around 500 kD in size, whereas the NUMAC complex possesses a size of approximately 1.5 MDa [25]. This finding additionally underlines that PRMT4 associates with other proteins in a cell type- and stimulus-dependent manner. The mass spectrometry identified Mi2α as a novel interaction partner of PRMT4 and subsequently we found that also its close homologue Mi2β is able to bind PRMT4. Both Mi2 proteins belong to the CHD family of chromatin remodellers containing a tandem Chromo domain and the SNF2-like ATPase domain as signature motifs [30]. They have predominantly been described in the literature as transcriptional repressors, since they are subunits of the NuRD repressor complex [34]–[36]. Several transcription factors, for example NF-κB and KRAB, have been shown to recruit the repressive activity of NuRD to target genes [54], [55]. Nevertheless, the two Mi2 proteins seem to have distinct function, since KRAB is specifically corepressed by NuRD containing Mi2α and not by Mi2β. We excluded the possibility that the PRMT4-Mi2 interaction takes place within the NuRD complex, since PRMT4 did not interact with two other subunits of NuRD and additionally the molecular weight of the PRMT4 complex separated via gel filtration was smaller than the expected size (∼1 MDa) of the NuRD complex [36].

PRMT4 acts as a coactivator of several transcription factors, particularly well-studied for ER, and requires for this function its methyltransferase activity, which marks active gene promoters by H3R17 methylation [7], [18]. We therefore envisaged a potential role of the PRMT4-Mi2 interaction in transcriptional activation, to which Mi2 proteins have also been functionally linked. It was found that Mi2β associates with essential transcription factors of lymphocyte development, such as Ikaros and HEB. Mi2β promotes *CD4* gene expression together with HEB and the HAT p300 in developing thymocytes [37], whereas Mi2β and Ikaros antagonise each other's silencing activity and thereby allow transcriptional activation of lymphocyte-specific genes [56], [57]. Mi2α was reported to coactivate transcription mediated by the transcription factor c-Myb [39]. Given that PRMT4, Mi2β and c-Myb consistently revealed defects in the development of specific haematopoietic lineages in knockout models [37], [42], [49], we focussed on the potential functional link of the PRMT4-Mi2 interaction in the context of c-Myb signalling. We found that PRMT4 and c-Myb bind each other and additionally c-Myb associates with both Mi2 proteins. When we studied the impact of PRMT4 and Mi2 in HD11 macrophage cells, a well-established model for c-Myb function, we detected that PRMT4 and Mi2 are recruited to the endogenous target gene *Mim-1* in c-Myb-dependent manner and cooperate in activation of c-Myb-mediated transcription. A recent report showed that among the C/EBP transcription factors, which are also known to activate *Mim-1*, C/EBPβ is antagonised by PRMT4-mediated arginine methylation [58]. However, in our experimental system the coactivating function of PRMT4 is predominant for the *Mim-1* gene regulation.

Interestingly, recruitment of PRMT4 and Mi2 was detected at the *Mim-1* promoter as well as enhancer in a c-Myb-dependent manner, whereas c-Myb preferentially bound to the promoter. Therefore, the recruitment of the two coactivators did not reflect the binding preference of c-Myb for the promoter, which might be due to c-Myb associations that differ in their composition at the enhancer and the promoter and lead to the masking of the antibody epitope within the c-Myb protein specifically when bound to the enhancer. Furthermore, other factors than c-Myb, with preferential binding to the enhancer, might promote binding of PRMT4 and Mi2 to the enhancer. Another interpretation of this observation is that after 30 hours of doxycycline treatment the chromatin structure has changed. Nucleosome repositioning, which has been described in the regulatory regions of the *mim-1* gene [59], might lead to a better access of c-Myb to its binding sites selectively in the promoter region and therefore stronger recruitment of c-Myb to the promoter compared to the enhancer. Although PRMT4 and Mi2 recruitment to the promoter and enhancer depends on c-Myb, directly or indirectly, the coactivators do not necessarily have to follow c-Myb in their binding strength.

For both Mi2 proteins, the isolated N-terminus was shown to activate transcription in reporter gene assays [39], [60], which is interesting, as we mapped the N-terminus together with the Chromo domains in Mi2 to be responsible for the interaction with PRMT4. Given that the tandem Chromo domains were shown to stimulate the ATPase function of Mi2 [61], association of PRMT4 through these domains might influence the remodelling activity. Furthermore, we show here that PRMT4 and Mi2 need their catalytic activity for coactivation of c-Myb, which is contrary to recent findings that Mi2α coactivates c-Myb in a helicase-independent fashion and that solely the repressive function of Mi2α requires the helicase activity [39]. Interestingly, the *Mim-1* gene undergoes intensive nucleosome remodelling at its enhancer region upon transcriptional activation in HD11 [59]. Whether nucleosome remodelling of c-Myb target genes is mediated by Mi2 will be subject of future studies. Our findings open up additional interesting questions e.g. of how PRMT4 and Mi2 mechanistically cooperate and whether Mi2 is a substrate of PRMT4.

c-Myb is predominantly expressed in immature haematopoietic cells and is involved in the regulation of proliferation and differentiation of stem cells and progenitor cells of the bone marrow, but also of colon and adult brain [49]. Several c-Myb target genes have been identified in the mammalian system in the past, the majority of which are activated by c-Myb and hint at its cell type-specific functions, i.e. cell cycle progression, differentiation and survival [49], [50]. Therefore we investigated whether PRMT4 and Mi2 are also relevant coactivators of c-Myb in the mammalian haematopoietic system. In these studies we used the CML-derived erythroleukemia cell line K562, since c-Myb has been shown to be functionally relevant in these cells [51]. We found that PRMT4 and Mi2 directly activated c-Myb target gene transcription. Depletion of PRMT4 or Mi2 resulted in deregulation of cell proliferation, apoptosis and erythrocyte differentiation resembling the effects caused by c-Myb depletion. These functional correlations are consistent with the physical interaction of the three proteins and their cooperation in gene expression and suggest for the first time a connection between PRMT4 and the Mi2α/β remodeller in c-Myb signalling and c-Myb-dependent erythropoiesis.

Elevated c-Myb levels due to overexpression or inappropriate activation by structural alterations of the protein sequence lead to a block in differentiation and contribute to the onset of certain human leukaemias, in particular AML, CML and T-ALL [49]. Recently, PRMT4 was identified in an shRNA screen among the group of genes required for disease maintenance in an AML mouse model, in which c-Myb is a critical driver of oncogenesis [62]. These findings together with our observations turn PRMT4 as well as Mi2 into attractive targets for cancer research and therapy in the future.

## Materials and Methods

### Cell lines and transfections

HEK293, HeLa, Molt-4, MCF-7 and U2OS cells were maintained in Dulbeccos Modified Eagle's Medium (Lonza), while K562 and Jurkat cells were cultured in RPMI 1640 (PAA). Growth medium was supplemented with 10% fetal calf serum (FCS, Invitrogen) and 1% Penicillin/Streptomycin (Lonza). HD11 and HD11-C3 (stably expressing a doxycycline-inducible chicken c-Myb) cells were cultured in Iscove's Medium (Biochrom AG) supplemented with 8% FCS and 2% chicken serum (Sigma). For induction of c-Myb expression, HD11-C3 cells were treated with 1 µg/ml doxycycline (Sigma) for 30 hours.

Transient transfections of HEK293 and HeLa cells with plasmids were performed following a standard CaPO_4_ protocol. HD11 cells were transiently transfected with plasmids using Fugene HD reagent (Roche). For siRNA transfection of K562 cells, 5×10^6^ cells were electroporated in 300 µl growth medium together with 6 µg siRNA (Dharmacon) at 220 V and 950 µF (Electroporator BioRad) using 4 mm cuvettes and subsequently cultured in growth medium. U2OS cells (1.6×10^5^ cells per 6-well) were transfected with 20 nM siRNA using Lipofectamin RNAiMax (Invitrogen).

### Plasmids, siRNAs, and antibodies

Short interfering RNA (siRNA) oligonucleotide duplexes were obtained from Dharmacon or Eurogentec. The siRNA sequences, the plasmids and antibodies used are listed in the Text S1.

### Protein extraction and immunoprecipitation (IP)

For whole-cell extracts, cells were lysed in IPH buffer (50 mM Tris at pH 8, 150 mM NaCl, 5 mM EDTA, 0.5% NP40, 1 mM DTT). To digest the cellular DNA, lysates were subjected to 62.5 units Benzonase (Invitrogen) per mg protein with addition of 7 mM MgCl_2_ for 40 min at 4°C. For co-immunoprecipitation (co-IP) assay, samples of 0.5–1 mg protein were adjusted to the same volume and to 10 µg/ml ethidium bromide. The IP procedure was performed according to [63].

### Gel filtration, ion exchange chromatography, and mass spectrometry

Gel filtration chromatography for determination of the native size of PRMT4 and ion exchange chromatography for purification of PRMT4 with associated proteins followed by IP and mass spectrometry are described in the Text S1.

### Recombinant proteins and GST-pulldown experiments

GST- and His-tagged proteins were purified from *E. coli* BL21 according to standard protocols. In vitro transcription and translation (IVT) of Mi2α deletion constructs in the presence of ^35^S-labelled methionine was performed with TnT T7 Coupled Reticulocyte Lysate System (Promega) according to the manufacturer's protocol. Between 0.1–10 µl of each IVT product and 1 µg GST-/His-tagged proteins were used per pulldown reaction. GST-pulldown experiments were performed as previously described [63]. The reactions were finally separated by SDS-PAGE and proteins were detected either by Western Blot analysis or autoradiography.

### Reverse transcription quantitative PCR (RT–qPCR) and chromatin immunoprecipitation quantitative PCR (ChIP–qPCR)

Total RNA was isolated using PeqGold total RNA Kit (PeqLab). First strand cDNA was synthesised from 0.5 µg of RNA by incubation with oligodT_17_ primer and 100 units M-MLV reverse transcriptase (Invitrogen) as described by the manufacturer. For chromatin immunoprecipitation (ChIP) analysis, a 145 cm^2^ dish of HD11 cells or 1×10^7^ K562 cells were used per IP. The protocol was carried out according to [12] except that chromatin was fragmented by sonication 50×3 sec and 5 sec pause on ice at 30% amplitude (Branson Sonifier W-250-D). cDNA and eluted chromatin were subjected to qPCR analysis in triplicates with gene-specific primers listed in the Text S1. Quantitative PCR was performed using Absolute qPCR SYBR Green Mix (Thermo Scientific) and the Mx3000P real-time detection system (Agilent). Each qPCR reaction was performed in triplicates from the same experiment (technical replicates) and the standard deviation (indicated by error bars) was calculated accordingly. The presented data sets are representative of at least 3 independent experiments (biological replicates). For RT-qPCR, each mRNA expression was normalised to *GAPDH* mRNA expression. ChIP-qPCR results were expressed as % input.

### FACS (fluorescence-activated cell sorting) analysis

For quantification of the cell cycle distribution, 1×10^6^ K562 cells were harvested 3 days after siRNA transfection, washed in PBS and fixed in ice-cold ethanol for 30 min. Cells were washed again in PBS and DNA was stained with 54 µM propidium iodide (PI) in the presence of 38 mM sodium citrate and 10 µg DNase-free RNase A (Applichem) in the dark for 30 min at 37°C. Samples were then analysed in a Flow Cytometer FACS Calibur using ModfitLT Mac3 Software and for sub-G1 using CellQuest-Pro software (BD Biosciences). Reproducible and representative data sets are shown.

### Colony formation assay

Three days after transfection of K562 cells with siRNA, 800 cells/100 µl growth medium were seeded in duplicates in 300 µl Methocult M2334 (Stem Cell Biotechnologies) supplemented with 20% RPMI and 1% Pen/Strep in 24-well plates. After 2 days of incubation, colonies were stained with 50 µl 1 mg/ml INT (Iodonitrotetrazolium chloride) for 5 days. Pictures of each well were captured with a binocular microscope (Leica MZ 125) using the Leica DC300 camera. Colonies larger than 0.1 mm were counted. Three independent experiments were analysed for quantification.

### Erythroid differentiation

Three days after transfection with siRNA, K562 cells were seeded in triplicates at a density of 4×10^5^ cells/ml and were treated for 3 days with 30 µM hemin [64]. Subsequently, 700 µl of the suspension cells were pelleted and washed twice with 0.9% NaCl solution. Then cells were resuspended in 100 µl 0.9% NaCl and 50 µl TMB solution (10 mg 3, 3′, 5, 5′-Tetramethyle-benzidine-dihydrochloride, 1.2 ml acetic acid, 8.8 ml ddH_2_O, 2% H_2_O_2_). After 30 min incubation, 200 µl 0.9% NaCl was added. The number of benzidine-positive cells was estimated by counting triplicates of 300 cells under a microscope using the Neubauer counting chamber.

## Supporting Information

Figure S1 Endogenous PRMT4 and Mi2α interact in the PRMT4-enriched MonoQ fractions. A: Validation of the anti-Mi2α serum in IP. HEK293 cells were transfected with Flag-Mi2α construct. Protein extracts were subjected to IP using anti-Mi2α serum or isotype control IgG (α-ctrl). Input (1%) and precipitates were stained by Western Blot analysis using anti-Flag antibody. B: PRMT4-enriched MonoQ fractions of HEK293 cells (Figure 1B) were incubated with anti-Mi2α (α-Mi2α) or control IgG (α-ctrl). Input (5%) and precipitates were stained by Western Blot analysis using anti-PRMT4 and anti-Mi2α antibodies.
(TIF)

Figure S2 Visualisation of GST and GST-PRMT fusion proteins by Coomassie staining. For GST-pulldown, GST and GST-PRMT proteins were purified from *E. coli*. The amounts of recombinant bead-bound proteins, which were employed in the assay, are visualised here together with 2 µg of BSA by Coomassie staining. The full-length protein bands are marked with asterisks.
(TIF)

Figure S3 Mi2 interacts with PRMT4 via its N-teminus and its tandem Chromo domain. A: Schematic representation of His-tagged Mi2α deletion constructs used in GST-pulldown assays. The conserved domains of the Mi2α/β subfamily are indicated in the full-length Mi2 protein. The deletion constructs are as the follows: N-terminal domain (Nt), paired PHD fingers (P), tandem Chromo domain (Ch), SNF2-like helicase domain (H) and a C-terminal domain (Ct). B: Equal amounts of bacterial purified recombinant GST and GST-PRMT4 were used for pulldown assay as visualised by Coomassie staining. Full-length GST-PRMT4 is marked with an asterisk. C: ^35^S-methionine-labelled Mi2α deletion constructs were synthesised by IVT. The reaction products were separated by SDS-PAGE and visualised by fluorography. D: Pulldown assays using 1 µg of glutathione bead-bound GST and GST-PRMT4, respectively (as in B) together with radiolabelled Mi2α deletion mutants (as in C) were performed. Bound proteins were separated by SDS-PAGE and visualised by fluorography. Signals of bound Mi2α deletion proteins are marked with asterisks.
(TIF)

Figure S4 PRMT4 does not interact with other subunits of the NuRD complex. HeLa cells were transfected with Flag-Mi2α construct (+) or empty vector (−). Protein extracts were subjected to IP using anti-PRMT4 (α-PRMT4), anti-Flag (α-Flag) or isotype control IgG (α-ctrl). Input (1%) and precipitates were stained by Western Blot analysis using anti-MBD3, anti-HDAC1 and anti-Flag antibodies.
(TIF)

Figure S5 Mi2α is an interaction partner of c-Myb. HEK293 cells were transfected with Flag-Mi2α, HA-c-Myb or empty vector (alone or in combination). Protein extracts were subjected to IP using anti-Flag (α-Flag). Input (0.5%) and precipitates were stained by Western Blot analysis using anti-Flag and anti-HA antibodies. The arrowhead indicates HA-c-Myb.
(TIF)

Figure S6 Overexpression of PRMT4 and Mi2α does not affect the expression levels of overexpressed c-Myb in HD11 cells. A, B: HD11 cells were transfected with the indicated constructs. After 48 hours, cells were harvested and protein extracts were subjected to Western Blot analysis using anti-Flag, anti-c-Myb, anti-HA and anti-β-Tubulin antibodies. The arrow indicates Flag-Mi2α (A) and Flag-Mi2β (B), respectively.
(TIF)

Figure S7 PRMT4 and Mi2α are synergistic coactivators of the c-Myb target gene *Lysozyme*. HD11 cells were transfected with the indicated constructs. After 48 hours, cells were harvested and total RNA was isolated. RT-qPCR was performed for detection of transcript levels of *Lysozyme*. Each mRNA expression was normalised to *GAPDH* mRNA expression. Transcript levels in empty vector-transfected cells (−) were set to 1.
(TIF)

Figure S8 Coactivation of the c-Myb-dependent target gene *Lysozyme* is specific for PRMT4. HD11 cells were transfected with the indicated constructs. After 48 hours, cells were harvested for total RNA isolation. Levels of *Lysozyme* mRNA were analysed by RT-qPCR and normalised to *GAPDH* mRNA levels. Transcript levels in empty vector-transfected cells (−) were set to 1.
(TIF)

Figure S9 The catalytic activity of PRMT4 and Mi2 is essential for their cooperative function on the *Lysozyme* gene activation. HD11 cells were transfected with tagged wild type (black bars) and catalytic mutant forms (grey bars) of PRMT4 and Mi2α (methyltransferase-dead PRMT4 mutant: VLD; helicase-dead Mi2α mutant: KA). 48 hours after transfection total RNA and protein extracts were isolated. Levels of *Lysozyme* mRNA were analysed by RT-qPCR and normalised to *GAPDH* mRNA levels. Transcript levels of empty vector-transfected cells (−) were set to 1. For detection of overexpression, protein levels of mutant and wild type PRMT4 and Mi2α were detected by Western Blot analysis using anti-Flag and anti-HA antibodies. β-Tubulin staining served as loading control.
(TIF)

Figure S10 Primary PI-FACS profiles of K562 cells depleted for c-Myb, PRMT4 and Mi2. K562 cells were transfected with siRNAs targeting c-Myb, PRMT4, Mi2α and Mi2β or with control siRNA (si ctrl). The DNA content of propidium iodide (PI)-stained cells was measured by flow cytometry (FACS). From each knockdown the percentage of cells in sub-G1, G1 and G2/M phase was determined. The primary FACS profiles analysed with ModfitLT Mac3 and percentage (%) of cells in each phase are depicted.
(TIF)

Figure S11 Cell cycle analysis of U2OS cells depleted for c-Myb, PRMT4 and Mi2. A: U2OS cells express low protein levels of c-Myb compared to K562 and Jurkat cells. Protein extracts (50 µg) of K562 and U2OS cells were subjected to Western Blot analysis using anti-c-Myb and anti-β-Tubulin antibodies. B: Knockdown efficiency of PRMT4, Mi2α and Mi2β in U2OS cells was determined. U2OS cells were transfected with the indicated siRNAs targeting PRMT4, Mi2α and Mi2β or with control siRNA (si ctrl). Cells were harvested 2 days later and total RNA was isolated. For detection of knockdown efficiency of *PRMT4*, *Mi2α* and *Mi2β* at mRNA levels, RT-qPCR was conducted using gene-specific primers and normalised to *GAPDH*. Transcript levels in si ctrl-treated cells were set to 1. C: PRMT4 and Mi2 depletion has different effects on apoptosis in U2OS cells than in K562 cells. Cells were treated as in B. Subsequently, the DNA content of PI-stained cells was measured by FACS. From each knockdown the percentage of cells in sub-G1, G1 and G2/M phase was determined. Shown are the changes in percentage (%) relative to the si ctrl condition. A representative data set is depicted.
(TIF)

Text S1 Supporting Material and Methods.
(DOC)
