# Supplementary figures and images for: PRMT4 Is a Novel Coactivator of c-Myb-Dependent Transcription in Haematopoietic Cell Lines
Source: PLoS Genet. 2013 Mar 7;9(3):e1003343. doi: 10.1371/journal.pgen.1003343 (PMC3591284; doi:10.1371/journal.pgen.1003343)

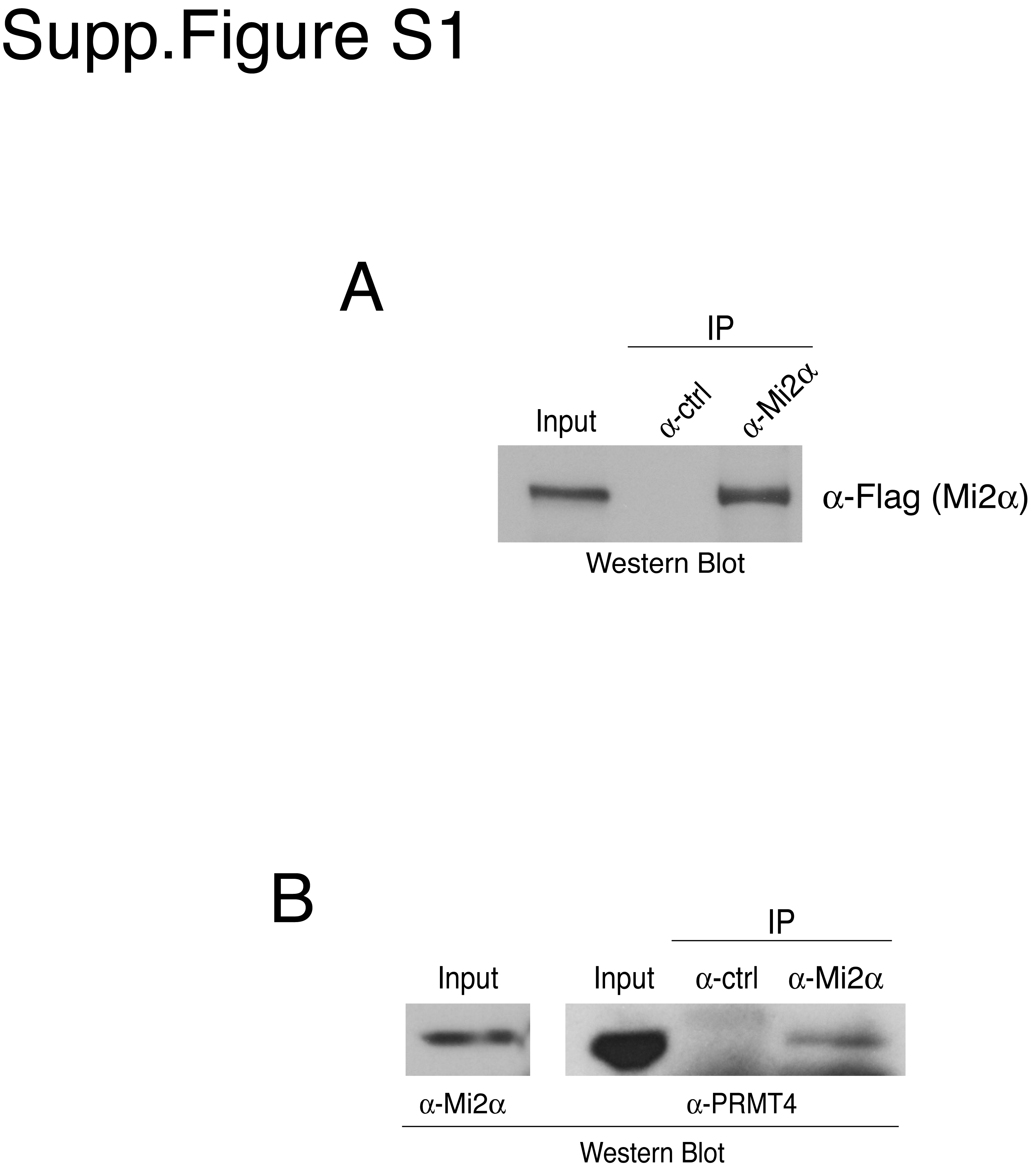

Supplement: Figure S1 — Endogenous PRMT4 and Mi2α interact in the PRMT4-enriched MonoQ fractions. A: Validation of the anti-Mi2α serum in IP. HEK293 cells were transfected with Flag-Mi2α construct. Protein extracts were subjected to IP using anti-Mi2α serum or isotype control IgG (α-ctrl). Input (1%) and precipitates were stained by Western Blot analysis using anti-Flag antibody. B: PRMT4-enriched MonoQ fractions of HEK293 cells (Figure 1B) were incubated with anti-Mi2α (α-Mi2α) or control IgG (α-ctrl). Input (5%) and precipitates were stained by Western Blot analysis using anti-PRMT4 and anti-Mi2α antibodies. (TIF) [file pgen.1003343.s001.tif]

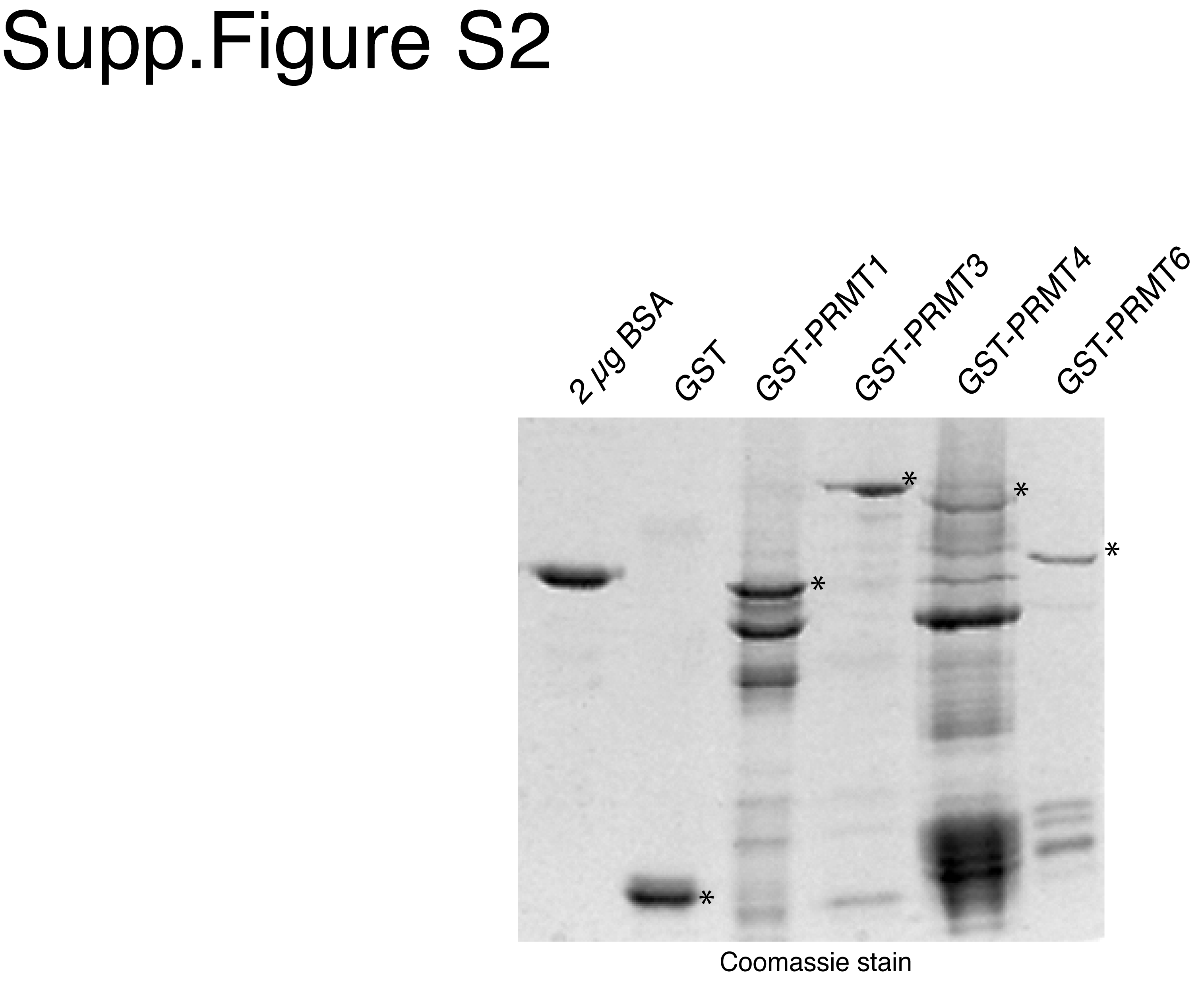

Supplement: Figure S2 — Visualisation of GST and GST-PRMT fusion proteins by Coomassie staining. For GST-pulldown, GST and GST-PRMT proteins were purified from E. coli. The amounts of recombinant bead-bound proteins, which were employed in the assay, are visualised here together with 2 µg of BSA by Coomassie staining. The full-length protein bands are marked with asterisks. (TIF) [file pgen.1003343.s002.tif]

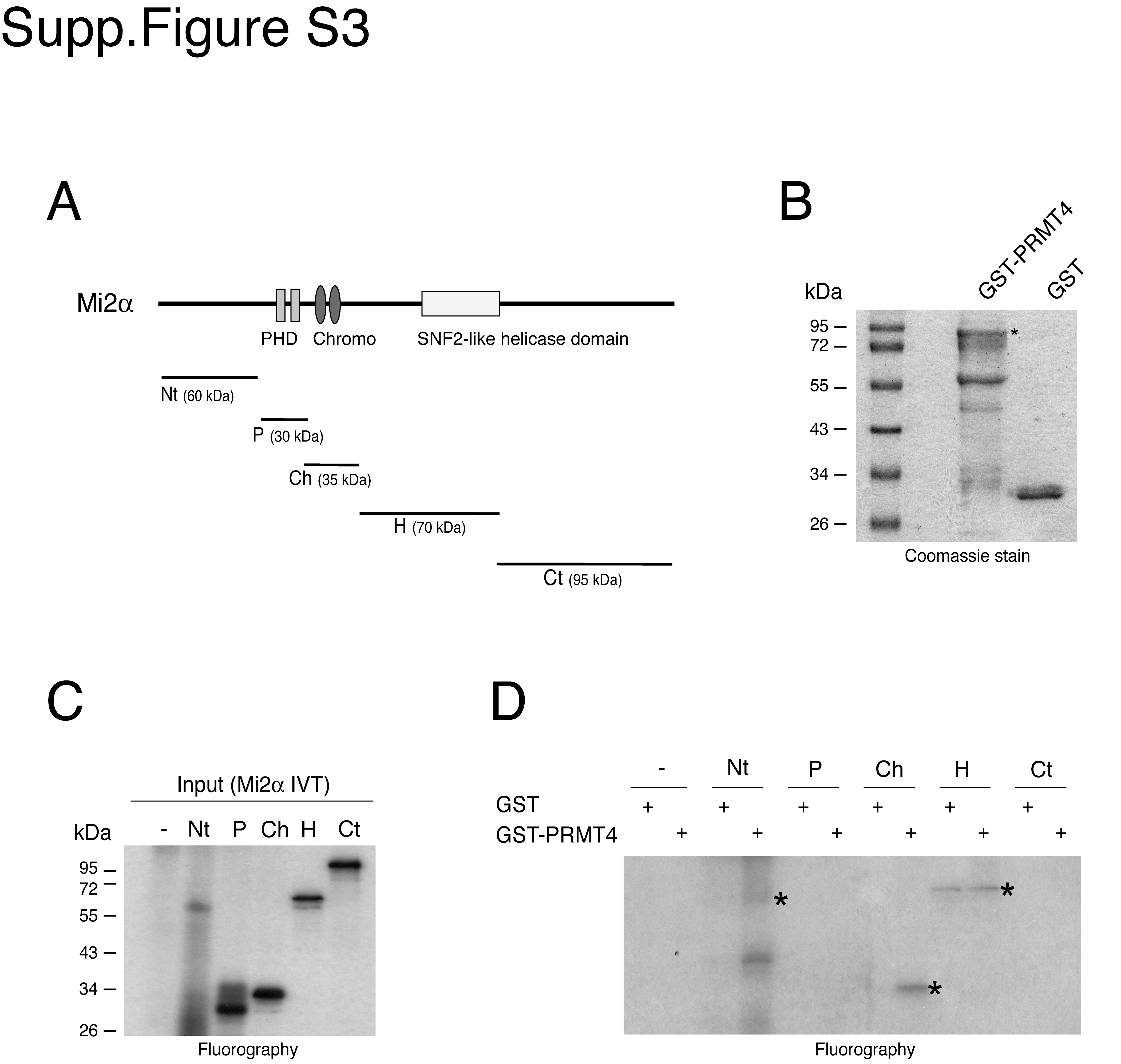

Supplement: Figure S3 — Mi2 interacts with PRMT4 via its N-teminus and its tandem Chromo domain. A: Schematic representation of His-tagged Mi2α deletion constructs used in GST-pulldown assays. The conserved domains of the Mi2α/β subfamily are indicated in the full-length Mi2 protein. The deletion constructs are as the follows: N-terminal domain (Nt), paired PHD fingers (P), tandem Chromo domain (Ch), SNF2-like helicase domain (H) and a C-terminal domain (Ct). B: Equal amounts of bacterial purified recombinant GST and GST-PRMT4 were used for pulldown assay as visualised by Coomassie staining. Full-length GST-PRMT4 is marked with an asterisk. C: 35S-methionine-labelled Mi2α deletion constructs were synthesised by IVT. The reaction products were separated by SDS-PAGE and visualised by fluorography. D: Pulldown assays using 1 µg of glutathione bead-bound GST and GST-PRMT4, respectively (as in B) together with radiolabelled Mi2α deletion mutants (as in C) were performed. Bound proteins were separated by SDS-PAGE and visualised by fluorography. Signals of bound Mi2α deletion proteins are marked with asterisks. (TIF) [file pgen.1003343.s003.tif]

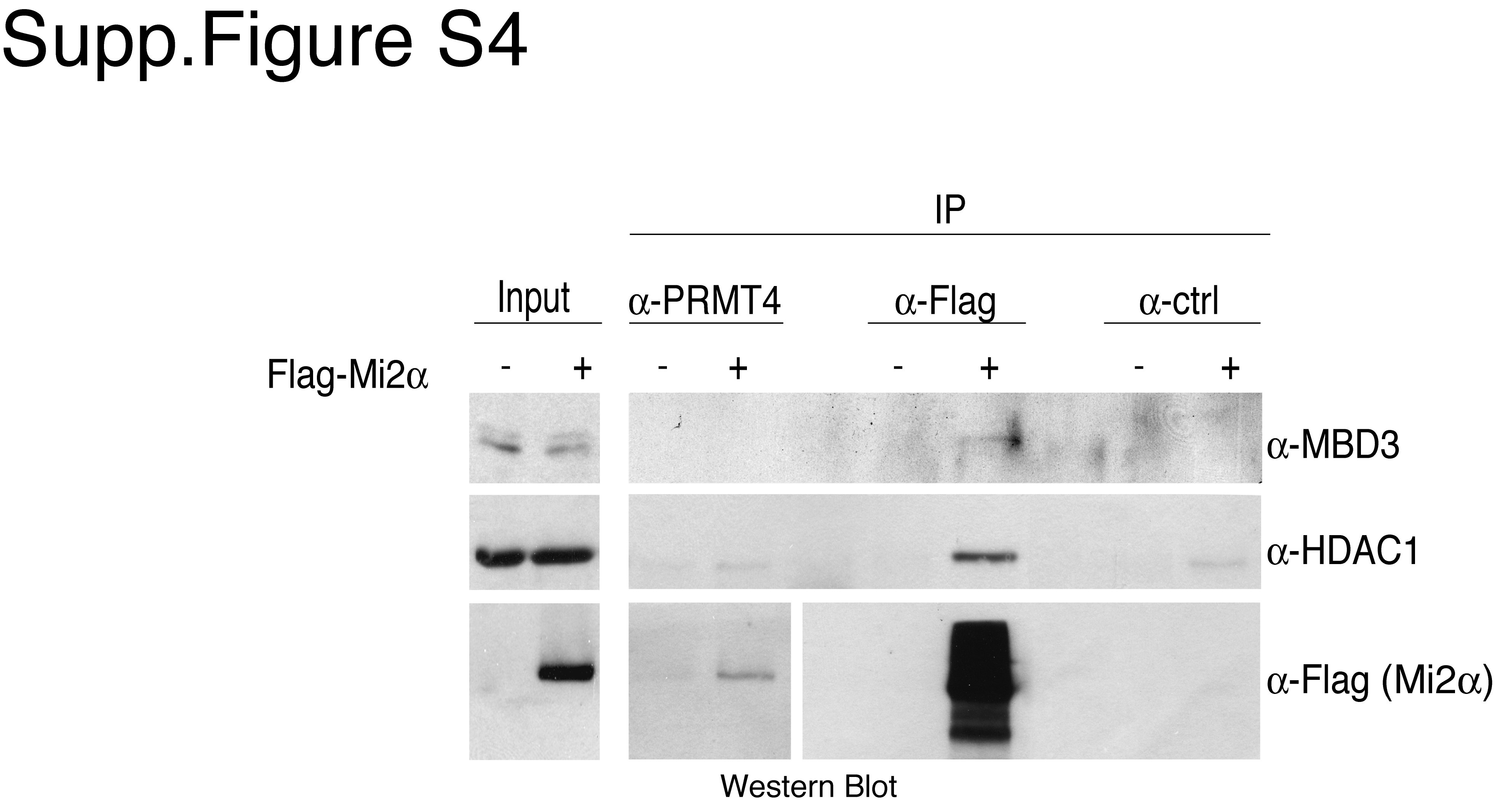

Supplement: Figure S4 — PRMT4 does not interact with other subunits of the NuRD complex. HeLa cells were transfected with Flag-Mi2α construct (+) or empty vector (−). Protein extracts were subjected to IP using anti-PRMT4 (α-PRMT4), anti-Flag (α-Flag) or isotype control IgG (α-ctrl). Input (1%) and precipitates were stained by Western Blot analysis using anti-MBD3, anti-HDAC1 and anti-Flag antibodies. (TIF) [file pgen.1003343.s004.tif]

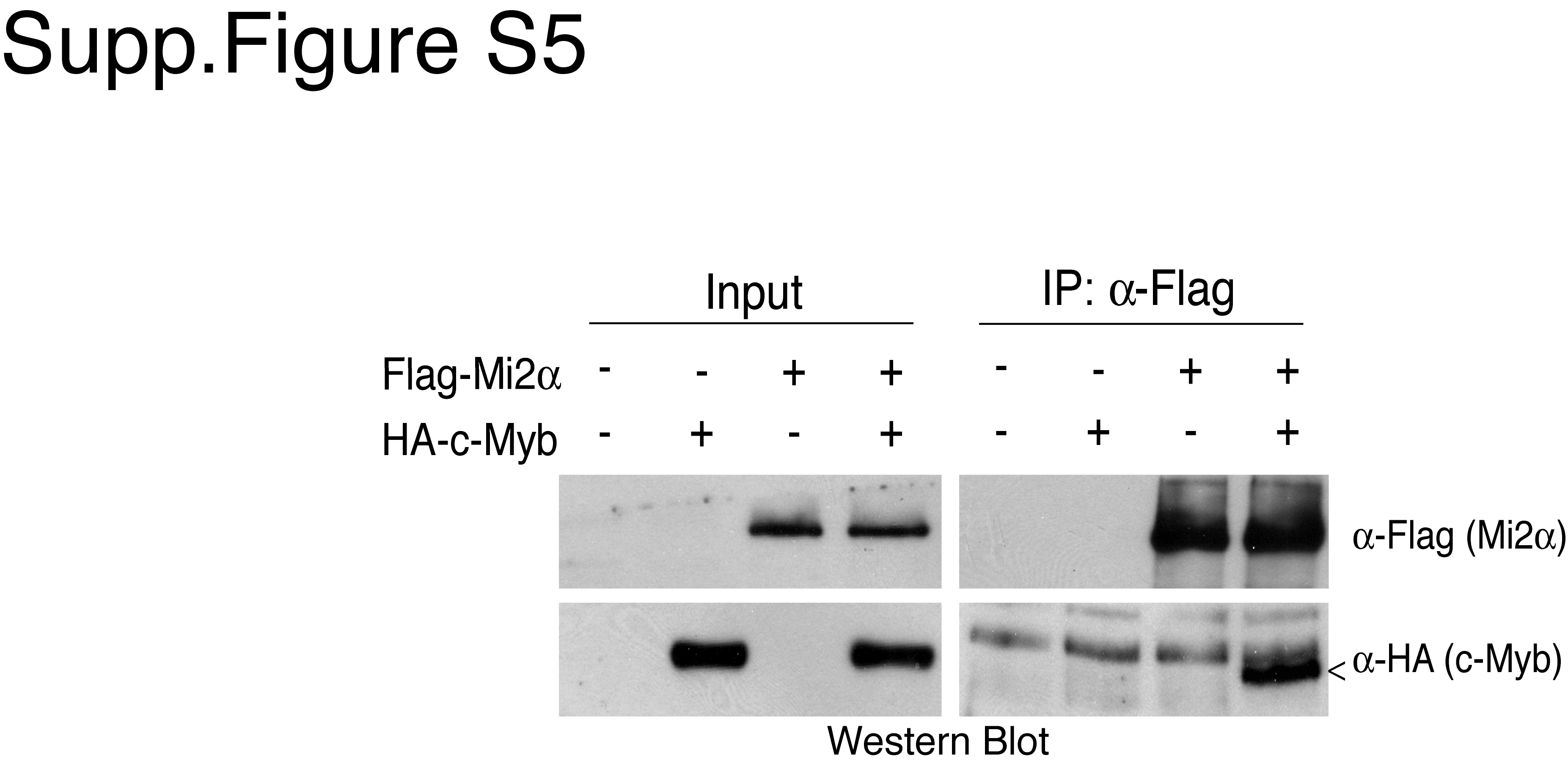

Supplement: Figure S5 — Mi2α is an interaction partner of c-Myb. HEK293 cells were transfected with Flag-Mi2α, HA-c-Myb or empty vector (alone or in combination). Protein extracts were subjected to IP using anti-Flag (α-Flag). Input (0.5%) and precipitates were stained by Western Blot analysis using anti-Flag and anti-HA antibodies. The arrowhead indicates HA-c-Myb. (TIF) [file pgen.1003343.s005.tif]

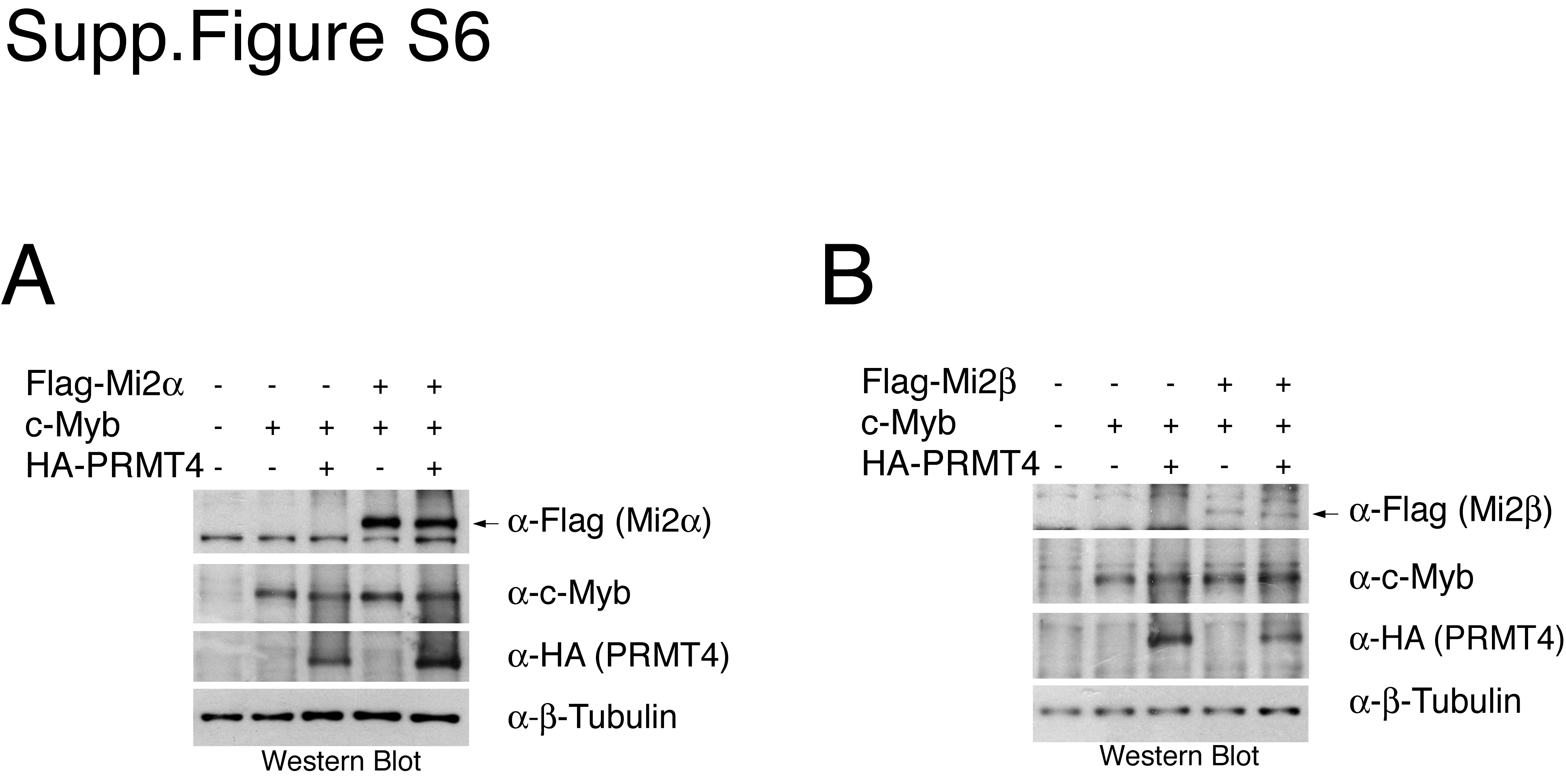

Supplement: Figure S6 — Overexpression of PRMT4 and Mi2α does not affect the expression levels of overexpressed c-Myb in HD11 cells. A, B: HD11 cells were transfected with the indicated constructs. After 48 hours, cells were harvested and protein extracts were subjected to Western Blot analysis using anti-Flag, anti-c-Myb, anti-HA and anti-β-Tubulin antibodies. The arrow indicates Flag-Mi2α (A) and Flag-Mi2β (B), respectively. (TIF) [file pgen.1003343.s006.tif]

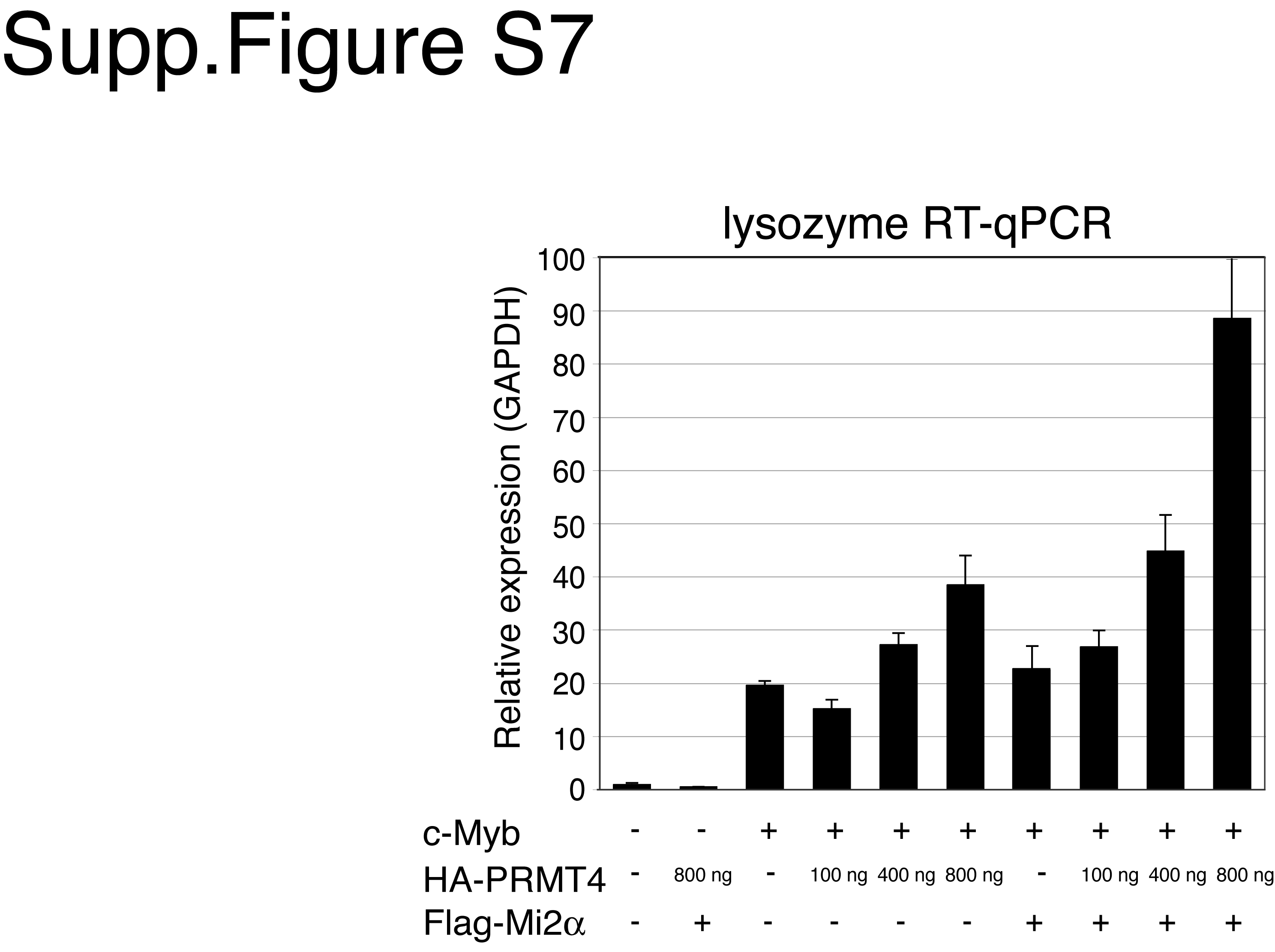

Supplement: Figure S7 — PRMT4 and Mi2α are synergistic coactivators of the c-Myb target gene Lysozyme. HD11 cells were transfected with the indicated constructs. After 48 hours, cells were harvested and total RNA was isolated. RT-qPCR was performed for detection of transcript levels of Lysozyme. Each mRNA expression was normalised to GAPDH mRNA expression. Transcript levels in empty vector-transfected cells (−) were set to 1. (TIF) [file pgen.1003343.s007.tif]

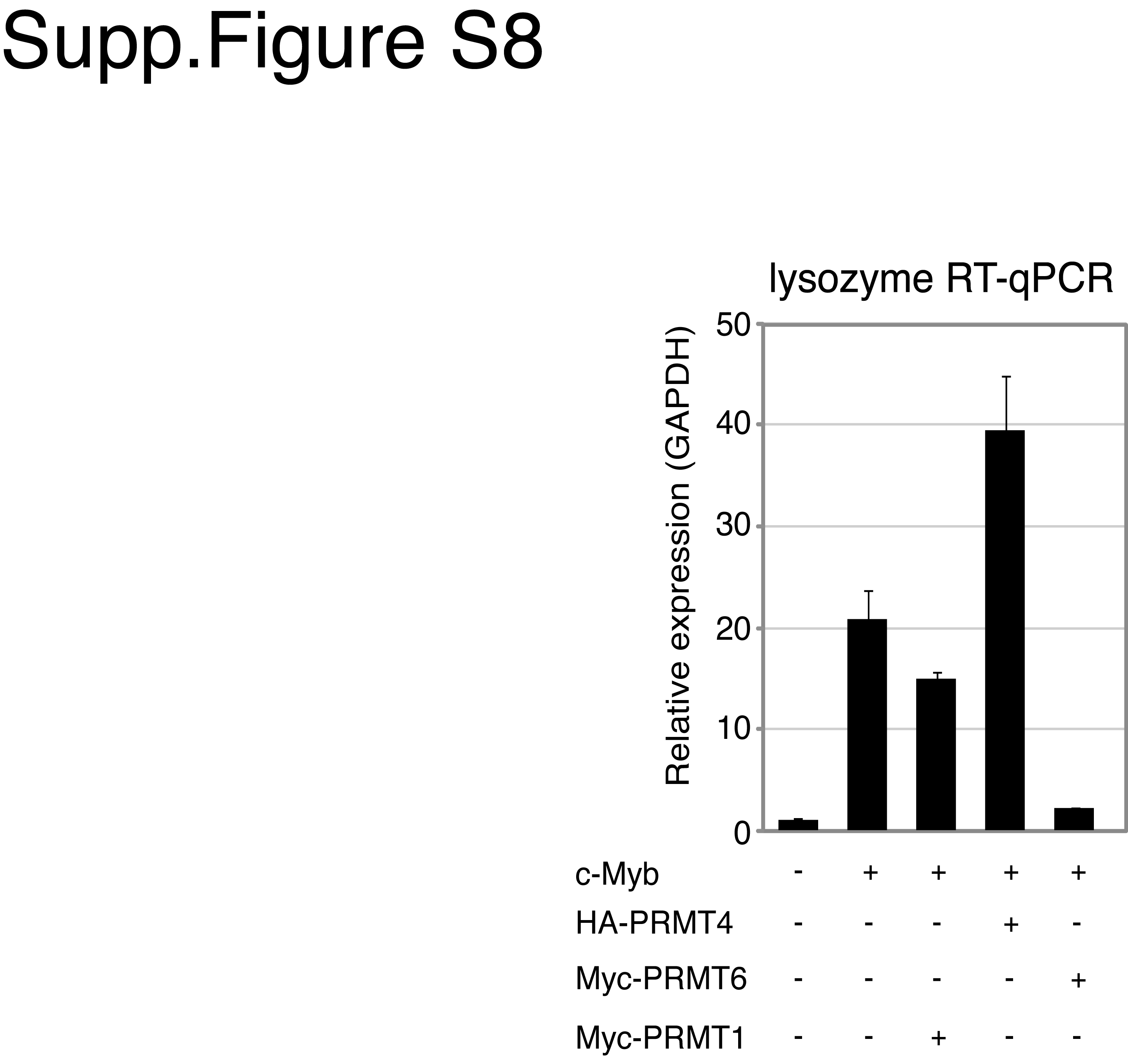

Supplement: Figure S8 — Coactivation of the c-Myb-dependent target gene Lysozyme is specific for PRMT4. HD11 cells were transfected with the indicated constructs. After 48 hours, cells were harvested for total RNA isolation. Levels of Lysozyme mRNA were analysed by RT-qPCR and normalised to GAPDH mRNA levels. Transcript levels in empty vector-transfected cells (−) were set to 1. (TIF) [file pgen.1003343.s008.tif]

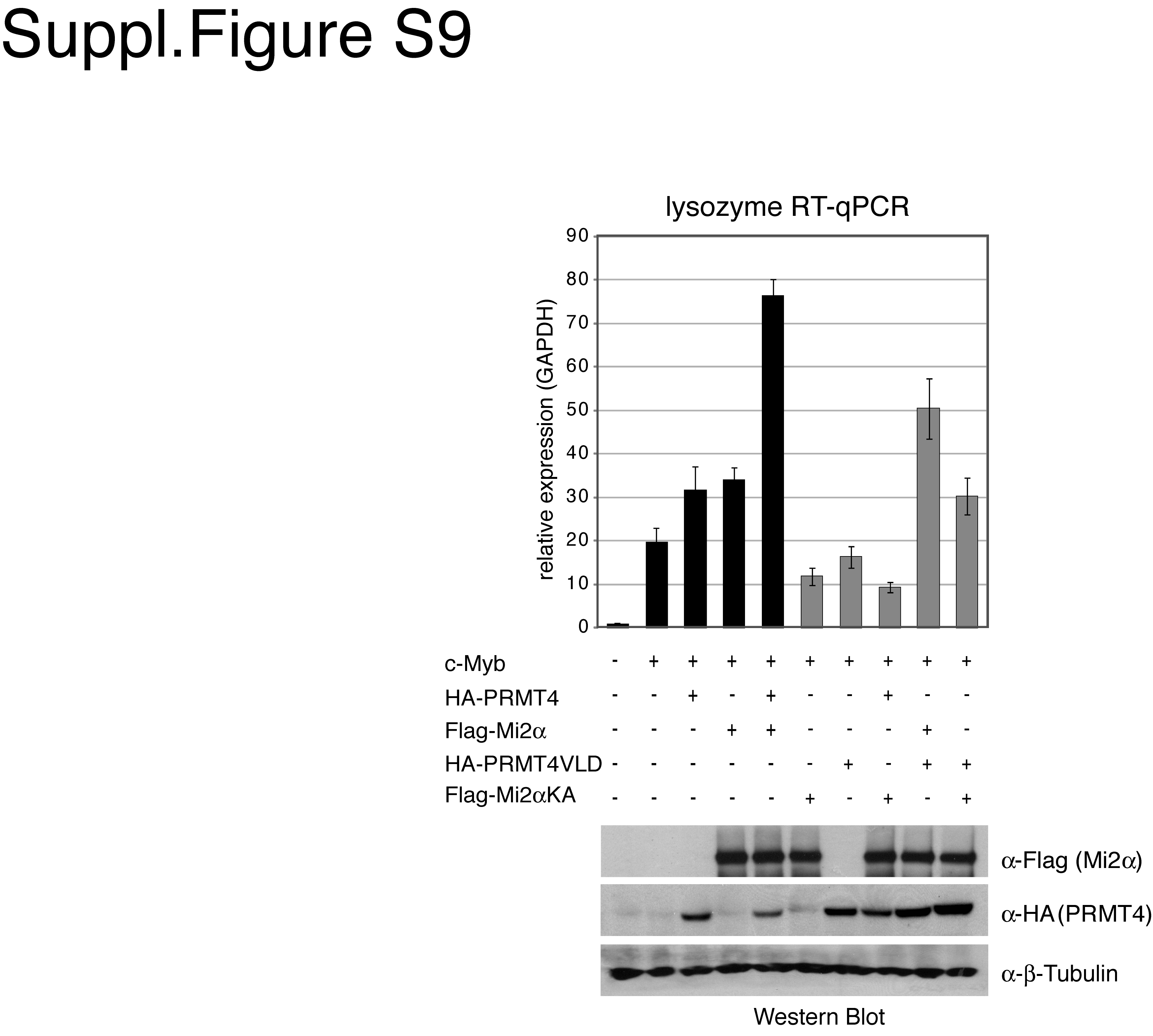

Supplement: Figure S9 — The catalytic activity of PRMT4 and Mi2 is essential for their cooperative function on the Lysozyme gene activation. HD11 cells were transfected with tagged wild type (black bars) and catalytic mutant forms (grey bars) of PRMT4 and Mi2α (methyltransferase-dead PRMT4 mutant: VLD; helicase-dead Mi2α mutant: KA). 48 hours after transfection total RNA and protein extracts were isolated. Levels of Lysozyme mRNA were analysed by RT-qPCR and normalised to GAPDH mRNA levels. Transcript levels of empty vector-transfected cells (−) were set to 1. For detection of overexpression, protein levels of mutant and wild type PRMT4 and Mi2α were detected by Western Blot analysis using anti-Flag and anti-HA antibodies. β-Tubulin staining served as loading control. (TIF) [file pgen.1003343.s009.tif]

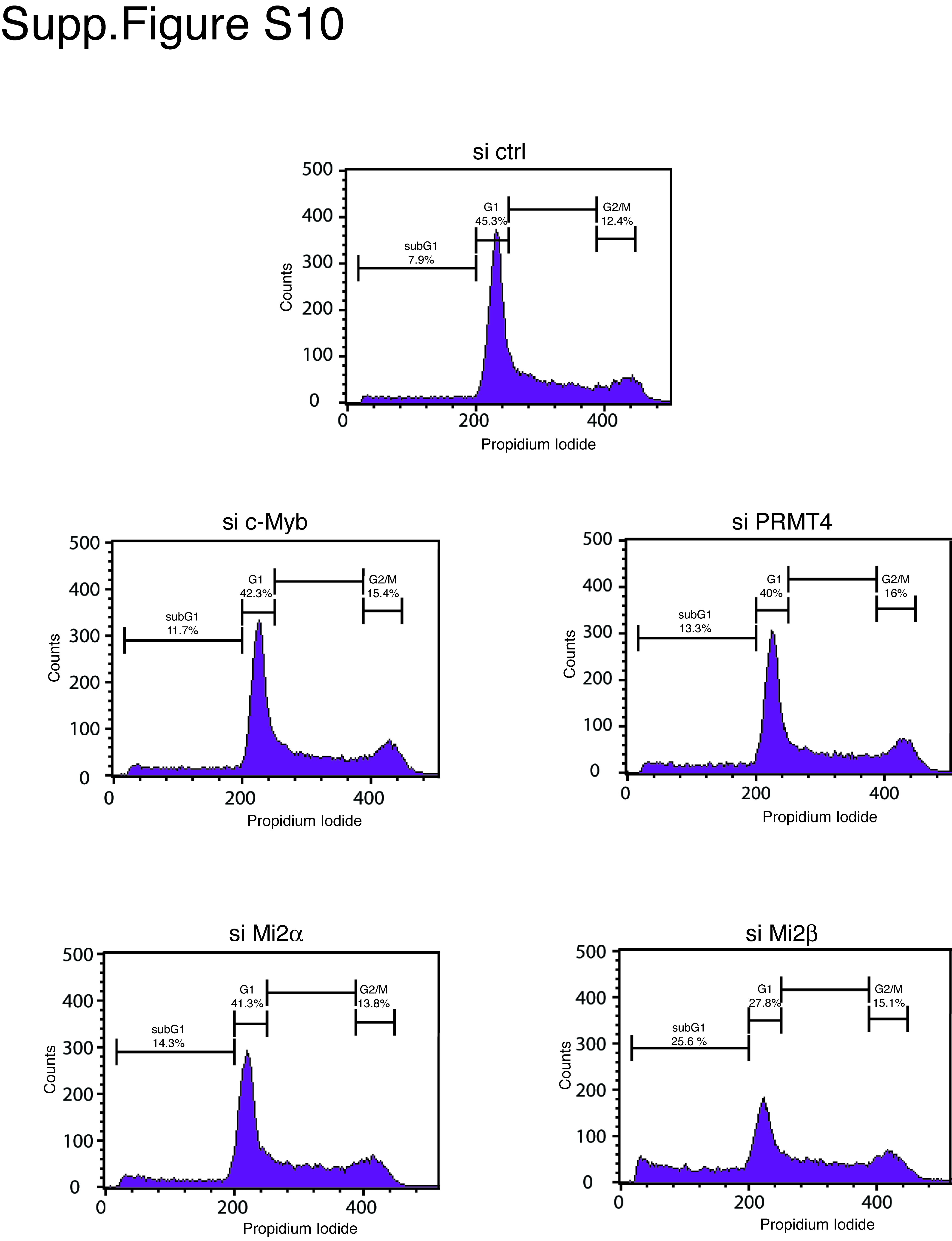

Supplement: Figure S10 — Primary PI-FACS profiles of K562 cells depleted for c-Myb, PRMT4 and Mi2. K562 cells were transfected with siRNAs targeting c-Myb, PRMT4, Mi2α and Mi2β or with control siRNA (si ctrl). The DNA content of propidium iodide (PI)-stained cells was measured by flow cytometry (FACS). From each knockdown the percentage of cells in sub-G1, G1 and G2/M phase was determined. The primary FACS profiles analysed with ModfitLT Mac3 and percentage (%) of cells in each phase are depicted. (TIF) [file pgen.1003343.s010.tif]

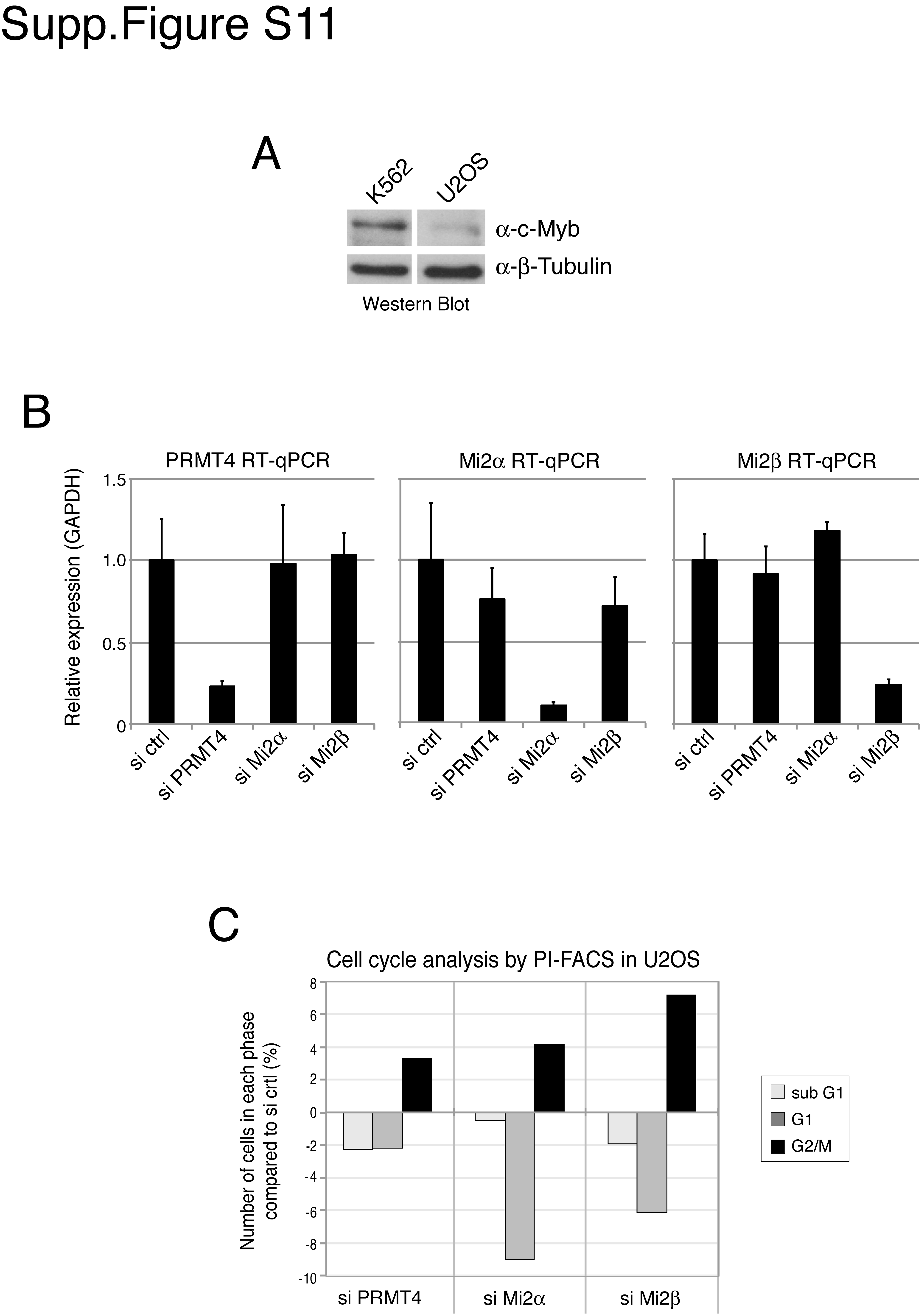

Supplement: Figure S11 — Cell cycle analysis of U2OS cells depleted for c-Myb, PRMT4 and Mi2. A: U2OS cells express low protein levels of c-Myb compared to K562 and Jurkat cells. Protein extracts (50 µg) of K562 and U2OS cells were subjected to Western Blot analysis using anti-c-Myb and anti-β-Tubulin antibodies. B: Knockdown efficiency of PRMT4, Mi2α and Mi2β in U2OS cells was determined. U2OS cells were transfected with the indicated siRNAs targeting PRMT4, Mi2α and Mi2β or with control siRNA (si ctrl). Cells were harvested 2 days later and total RNA was isolated. For detection of knockdown efficiency of PRMT4, Mi2α and Mi2β at mRNA levels, RT-qPCR was conducted using gene-specific primers and normalised to GAPDH. Transcript levels in si ctrl-treated cells were set to 1. C: PRMT4 and Mi2 depletion has different effects on apoptosis in U2OS cells than in K562 cells. Cells were treated as in B. Subsequently, the DNA content of PI-stained cells was measured by FACS. From each knockdown the percentage of cells in sub-G1, G1 and G2/M phase was determined. Shown are the changes in percentage (%) relative to the si ctrl condition. A representative data set is depicted. (TIF) [file pgen.1003343.s011.tif]
