# Supplementary material for: PRMT4 Is a Novel Coactivator of c-Myb-Dependent Transcription in Haematopoietic Cell Lines
Source: PLoS Genet. 2013 Mar 7;9(3):e1003343. doi: 10.1371/journal.pgen.1003343 (PMC3591284; doi:10.1371/journal.pgen.1003343)
Supplement: Text S1 — Supporting Material and Methods. (DOC) [file pgen.1003343.s012.doc]

**Text S1 (Supporting Material and Methods)**

**PRMT4 is a novel coactivator of c-Myb-dependent transcription in haematopoietic cell lines.**

Gundula Streubel, Caroline Bouchard, Hannah Berberich, Marc Zeller, Sophia Teichmann, Jürgen Adamkiewicz, Rolf Müller, Karl-Heinz Klempnauer, Uta-Maria Bauer

**Supporting Material and Methods**

**SiRNA sequences**

The sense strands of the siRNA sequences used in the manuscript are indicated below. Control knockdown was performed using a pool (si ctrl) of 3 control siRNA (siNON, siScrambled, siLuciferase). The c-Myb knockdown was performed using a pool of the 4 indicated siRNAs.

si NON 5’-UUG AUG UGU UUA GUC GCU A-3’

si Luciferase 5’-GAU UAU GUC CGG UUA UGU-3’

si Scrambled 5'- UAG CGA CUA AAC ACA UCA A-3’

si PRMT4_1 5’-CAU GAU GCA GGA CUA CGU G-3’

si PRMT4_2 5’- GGA CAU GUC UGC UUA UUG C-3’

si PRMT4_3 5'- UGG AGC CGG AUC UAA GAU G-3’

si c-Myb_1 5’- CCG AAA CGU UGG UCU GUU A-3’

si c-Myb_2 5’- CAA CAC CAU UUC AUA GAG A-3’

si c-Myb_3 5’-CAA CGA CUA UUC CUA UUA C-3’

si c-Myb_4 5’-GAA AUA CGG UCC GAA ACG U-3’

# si Mi2α_1 5’-CGU AUG AGC UGA UCA CCA U-3’

si Mi2α_2 5’-GAG GAG AAG UAC UAU CGU U-3’

# si Mi2β_1 5’- CCA AGG ACC UGA AUG AUG A -3’

si Mi2β_2 5’- CAA AGG UGC UGC UGA UGU A-3’

**Plasmids**

The following plasmids were used for bacterial expression and IVT: Empty vector pGEX2TK (GE Healthcare Life Science), pGex4T-1-human PRMT1 [1], pGex2T-rat PRMT3 [2], pGex4T-1-murine PRMT4 [3] and pGex2TK-human PRMT6 [4]. For bacterial expression of His-tagged human c-Myb, the cDNA fragment containing the ORF (aa 1-636) was generated by SalI (blunted)/XhoI restriction of pCI-neo-hc-Myb [5] and cloned into pRSET via EcoRI (blunted)/XhoI sites. Human Mi2α deletion constructs pRSET-Mi2α_Nt (aa 1-377), pRSET-Mi2α_P (aa 369-517), pRSET-Mi2α_Ch (aa 484-686), pRSET-Mi2α_H (aa 678-1245), pRSET-Mi2α_Ct (aa 1240-2000) were amplified by PCR from pCIneoBFlag-Mi2α [5] and cloned via BamHI/Bsp119I into pRSET. For eukaryotic expression, the following plasmids were used: Empty vectors pcDNA3GFP, pcDNA3HA, pcDNA3.1 (Invitrogen) and pSG5HA, the plasmids pcDNA3.1-human PRMT1 [6], pcDNA3.1-human PRMT6 [4], pSG5HA-murine PRMT4 and catalytically inactive version pSG5HA-murine PRMT4VLD [3], pDNA3-murine PRMT4 (cloned from pSG5HA-PRMT4 by restriction with EcoRI), pCIneoBFlag-Mi2α and helicase-deficient version pCIneoBFlag-Mi2αK767A [5], pcDNA3-Flag-human Mi2β (aa 1-1912) [7], pcDNA3-HA-human Mi2β (aa 1-1612, C-terminally truncated) [8], pCIneoB-human c-Myb-HA (aa 1-640) [9].

**Antibodies**

The following antibodies were applied: anti-Flag (F3165, Sigma), anti-HA.11 (MMS-101P, Covance), anti-c-Myb (clone1_1, Upstate), anti-β-Tubulin (Chemicon), anti-Mi2 (sc-11378, Santa Cruz) and rabbit serum against aa 1919-2000 of Mi2α, anti-HDAC1 (sc-8410, Santa Cruz), anti-MBD3 (sc-9402, Santa Cruz), anti-PRMT4 (sc-5421, Santa Cruz), anti-PRMT4 (against recombinant murine His-tagged PRMT4 protein aa 433–608) generated in rabbits and affinity-purified against GST-tagged PRMT4, anti-H3R17me2a (ab 8284, Abcam) and as control antibodies anti-p15 (sc-1429, Santa Cruz), rabbit IgG (31.235, Pierce and I5006, Sigma).

**Gel filtration chromatography, ion exchange chromatography and mass spectrometry**

HEK cell extract was dialysed against buffer 1 (20 mM Hepes pH 7.9, 20% (v/v) glycerol, 3 mM DTT, 0.2 M PMSF) containing 100 mM KCl and applied to phosphocellulose (Whatman, P11) at 10 mg of protein/ml bed volume. The flow-through was collected (PCA) and the column was washed sequentially in a stepwise fashion using buffer 1 containing 350 mM KCl (PCB), 500 mM KCl (PCC), 1.0 M KCl (PCD). The fractions were analysed by Western Blot. The PRMT4 immunoreactive PCA fraction was adjusted to 50 mM KCl with buffer 2 (20 mM Tris pH 8, 10% glycerol, 0.5 mM DTT, 1 mM PMSF). The PCA fraction was passed through a 30 ml DEAE-Fractogel 650 XK16 column (Merck, Darmstadt) at a capacity of 10 mg/ml. The column was washed with buffer 2 containing 50 mM NaCl. Proteins were eluted with an increasing NaCl gradient (50-180 mM NaCl in 64 ml) in 8 ml fractions and a 450 mM NaCl step. The majority of immunoreactive PRMT4 was found in the 80-130 mM NaCl fractions. These fractions were pooled and adjusted to 50 mM NaCl with buffer 2. The DEAE fractions were applied to an 8 ml MonoQ HR10/10 column (GE Healthcare) at a capacity of 5 mg/ml. The column was washed with buffer 2 containing 50 M NaCl. Proteins were eluted with an increasing NaCl gradient (50-250 mM NaCl in 40 ml) in 2 ml fractions and a final 1 M NaCl step. The majority of immunoreactive and catalytic active PRMT4 was found in the 125-200 mM NaCl fractions. These MonoQ fractions were pooled and applied to control IP or IP using affinity-purified anti-PRMT4 antibody in IPH-buffer. Precipitates were bound to Protein A/G-beads. Subsequently, proteins were separated by SDS-PAGE and subjected to silver staining. Protein bands of interest were cut out, digested with trypsin and analysed by peptide mass fingerprint using an Autoflex MALDI-TOF mass spectrometer (Bruker). Peptide mass lists were analysed in the Mascot server using the Swiss-Prot protein database [10].

For gel filtration (size exclusion) chromatography, 250 µg protein extract (in 100 µl) were applied to a Superose 6 or Superdex 200 HR10/30 column (GE Healthcare) using running buffer (20 mM Tris pH 8, 10% glycerol, 175 mM NaCl, 0.5 mM DTT and 1 mM PMSF). Fractions with a volume of 1 ml or 0.5 ml were collected. Columns were calibrated using BSA, IgG, ferritin, thyroglobulin and dextran blue.

**Primer sequences used in RT-qPCR and ChIP-qPCR**

For RT-qPCR of human sequences, the following primers were used:

hCdc7 forward 5’-TGC TAT GCA ACA GAT AAA GTT TGT AG-3’

reverse 5’-TCC TGG TGT ACC TGC CCT A-3’

hCyclinB1 forward 5’-CAT GGT GCA CTT TCC TCC TT-3’

reverse 5’-AGG TAA TGT TGT AGA GTT GGT GTCC-3’

hGAPDH forward 5’-AGC CAC ATC GCT CAG ACA C-3’

reverse 5’-G CCC AAT ACG ACC AAA TCC-3’

hGata3 forward 5’-CTC ATT AAG CCC AAG CGA AG-3’

reverse 5’-TCT GAC AGT TCG CAC AGG AC-3’

hMi2α forward 5’-GGG GCA AGA CTG AGA AGG A-3’

reverse 5’-CAT CGG CAA AGG TTT CAG AG-3’

hMi2β forward 5’-GGA GAC AGA GCC CAA AGG T-3’

reverse 5’-GGG GTC AGA TCT ATT GCT GAC TT-3’

hc-Myc forward 5’-CAC CAG CAG CGA CTC TGA-3’

reverse 5’-GAT CCA GAC TCT GAC CTT TTG C-3’

hPRMT4 forward 5’-CAC ACC GAC TTC AAG GAC AA-3’

reverse 5’-AAA AAC GAC AGG ATC CCA GA-3’

For RT-qPCR of *Gallus gallus* sequences, the following primers were used:

ggGAPDH forward 5’-CCT CTG GGA AGC TGT GGA-3’

reverse 5’-TTA GCA GCC CCA GTG GAC-3’

ggMim-1 forward 5’-GGT AAA GGA GAA AAG CAC AAG G-3’

reverse 5’-CAC CAG AAA AGG GAG CAT ACA-3’

ggLysozyme forward 5’-CCG ACT GAA TCC TAC AGA-3’

reverse 5’-ACG GGA TGT TGC ACA GGT-3’

For ChIP-qPCR, the following primers were used and amplicon locations are indicated with respect to the TSS:

hCyclin B1 promoter region (-17 until +219 bp):

forward 5’-ATC GCC CTG GAA ACG CAT TCT CT-3’

reverse 5’-AGA AGC AGA ACA CCG GAG GC-3’

hCyclin B1 control region (-4633 until -4569 bp):

forward 5’-GAG GGC TGA AGG AGG GAT AG-3’

reverse 5’-CTG CAC CCA TCA ACT CGT C-3’

hCdc7 promoter region (-555 until -471 bp):

forward 5’- CCG TTA TTG TCA TCG CTT CA-3’

reverse 5’- TTT TCT AGG GGC TGG AAT CA-3’

hCdc7 control region (-7296 bp until -7223 bp):

forward GCT CAC TGT AGC CTC GAC CT-3’

reverse CCT CCA GTC CCA GCT ACT TG-3’

hc-Myc, promoter region (-1217 until -1082 bp)

forward 5’-AAA AGG GGA AAGAGG ACC TGG-3’

reverse 5’-CCT AAA AGG GGC AAG TGG AGA G-3’

hc-Myc control region (-3769 until -3685 bp):

forward 5’-TAGAGATGGCACGTC ACC AC-3’

reverse 5’-GCA CTT TGG GAG GCT CAA-3’

hβ-Tubulin promoter region (-448 until -152 bp)

forward 5’-TGT CTT CCC CAC CAC CCA ACT-3’

reverse 5’-CAA AGC CTC ATC GAG CCT GGC-3’

ggMim-1 promoter region (-201 until -75 bp)

forward 5’-acc tgt ctt tcc caa cca gct cta-3’

reverse 5’-ctg ttg tgt tgg cca atc agt cct-3’

ggMim-1 enhancer region (-2022 until -1916 bp)

forward 5’-aat gca gga atc cca cca gca ttg-3’

reverse 5’-tgg tggttg agg ctt ctC AGT Tca-3’

ggMim-1 upstream control region (-6687 until -6544 bp)

forward 5’- Aaggcaataaggggtgctct-3’

reverse 5’-ctttgtaagcagcatgcagc-3’

**References of Text S1**

1. Scott, H.S., Antonarakis, S.E., Lalioti, M.D., Rossier, C., Silver, P.A., et al. (1998) Identification and characterization of two putative human arginine methyltransferases (HRMT1L1 and HRMT1L2). Genomics 48: 330-340.

2. Tang, J., Gary, J.D., Clarke, S. and Herschman, H.R. (1998) PRMT 3, a type I protein arginine N-methyltransferase that differs from PRMT1 in its oligomerization, subcellular localization, substrate specificity, and regulation. J Biol Chem 273: 16935-16945.

3. Chen, D., Ma, H., Hong, H., Koh, S.S., Huang, S.M., et al. (1999) Regulation of transcription by a protein methyltransferase. Science 284: 2174-2177.

4. Hyllus, D., Stein, C., Schnabel, K., Schiltz, E., Imhof, A., et al. (2007) PRMT6-mediated methylation of R2 in histone H3 antagonizes H3 K4 trimethylation. Genes Dev 21: 3369-3380.

5. Saether, T., Berge, T., Ledsaak, M., Matre, V., Alm-Kristiansen, A.H., et al. (2007) The chromatin remodeling factor Mi-2alpha acts as a novel co-activator for human c-Myb. J Biol Chem 282: 13994-14005.

6. Balint, B.L., Szanto, A., Madi, A., Bauer, U.M., Gabor, P., et al. (2005) Arginine methylation provides epigenetic transcription memory for retinoid-induced differentiation in myeloid cells. Mol Cell Biol 25: 5648-5663.

7. Feng, Q. and Zhang, Y. (2001) The MeCP1 complex represses transcription through preferential binding, remodeling, and deacetylating methylated nucleosomes. Genes Dev 15: 827-832.

8. Shimono, Y., Murakami, H., Kawai, K., Wade, P.A., Shimokata, K., et al. (2003) Mi-2 beta associates with BRG1 and RET finger protein at the distinct regions with transcriptional activating and repressing abilities. J Biol Chem 278: 51638-51645.

9. Dahle, O., Andersen, T.O., Nordgard, O., Matre, V., Del Sal, G., et al. (2003) Transactivation properties of c-Myb are critically dependent on two SUMO-1 acceptor sites that are conjugated in a PIASy enhanced manner. Eur J Biochem 270: 1338-1348.

10. Adamkiewicz, J., Kaddatz, K., Rieck, M., Wilke, B., Muller-Brusselbach, S., et al. (2007) Proteomic profile of mouse fibroblasts with a targeted disruption of the peroxisome proliferator activated receptor-beta/delta gene. Proteomics 7: 1208-1216.
